# De novo transcriptomic subtyping of colorectal cancer liver metastases in the context of tumor heterogeneity

**DOI:** 10.1186/s13073-021-00956-1

**Published:** 2021-09-01

**Authors:** Seyed H. Moosavi, Peter W. Eide, Ina A. Eilertsen, Tuva H. Brunsell, Kaja C. G. Berg, Bård I. Røsok, Kristoffer W. Brudvik, Bjørn A. Bjørnbeth, Marianne G. Guren, Arild Nesbakken, Ragnhild A. Lothe, Anita Sveen

**Affiliations:** 1 Department of Molecular Oncology, Institute for Cancer Research, Oslo University Hospital, P.O. box 4953 Nydalen, NO-0424 Oslo, Norway; 2 K.G. Jebsen Colorectal Cancer Research Centre, Division for Cancer Medicine, Oslo University Hospital, P.O. Box 4953 Nydalen, NO-0424 Oslo, Norway; 3 Institute of Clinical Medicine, Faculty of Medicine, University of Oslo, P.O. box 1171 Blindern, NO-0318 Oslo, Norway; 4 Department of Gastrointestinal Surgery, Oslo University Hospital, P.O. box 4950, NO-0424 Oslo, Norway; 5 Department of Oncology, Oslo University Hospital, P.O. box 4953, NO-0424 Oslo, Norway

**Keywords:** Colorectal cancer, Liver metastasis, Transcriptomic subtyping, Gene expression profiling, Gene set enrichment analyses, Metastatic heterogeneity, Prediction models, Prognostic factor

## Abstract

**Background:**

Gene expression-based subtyping has the potential to form a new paradigm for stratified treatment of colorectal cancer. However, current frameworks are based on the transcriptomic profiles of primary tumors, and metastatic heterogeneity is a challenge. Here we aimed to develop a de novo metastasis-oriented framework.

**Methods:**

In total, 829 transcriptomic profiles from patients with colorectal cancer were analyzed, including primary tumors, liver metastases, and non-malignant liver samples. High-resolution microarray gene expression profiling was performed of 283 liver metastases from 171 patients treated by hepatic resection, including multiregional and/or multi-metastatic samples from each of 47 patients. A single randomly selected liver metastasis sample from each patient was used for unsupervised subtype discovery by nonnegative matrix factorization, and a random forest prediction model was trained to classify multi-metastatic samples, as well as liver metastases from two independent series of 308 additional patients.

**Results:**

Initial comparisons with non-malignant liver samples and primary colorectal tumors showed a highly variable degree of influence from the liver microenvironment in metastases, which contributed to inter-metastatic transcriptomic heterogeneity, but did not define subtype distinctions. The de novo liver metastasis subtype (LMS) framework recapitulated the main distinction between epithelial-like and mesenchymal-like tumors, with a strong immune and stromal component only in the latter. We also identified biologically distinct epithelial-like subtypes originating from different progenitor cell types. LMS1 metastases had several transcriptomic features of cancer aggressiveness, including secretory progenitor cell origin, oncogenic addictions, and microsatellite instability in a microsatellite stable background, as well as frequent *RAS*/*TP53* co-mutations. The poor-prognostic association of LMS1 metastases was independent of mutation status, clinicopathological variables, and current subtyping frameworks (consensus molecular subtypes and colorectal cancer intrinsic subtypes). LMS1 was also the least heterogeneous subtype in comparisons of multiple metastases per patient, and tumor heterogeneity did not confound the prognostic value of LMS1.

**Conclusions:**

We report the first large study of multi-metastatic gene expression profiling of colorectal cancer. The new metastasis-oriented subtyping framework showed potential for clinically relevant transcriptomic classification in the context of metastatic heterogeneity, and an LMS1 mini-classifier was constructed to facilitate prognostic stratification and further clinical testing.

**Supplementary Information:**

The online version contains supplementary material available at 10.1186/s13073-021-00956-1.

## Background

Gene expression profiles of colorectal cancers (CRCs) have strong clinical associations. Prognostic value has consistently been shown for signatures of immune and stromal cells infiltrating the tumor microenvironment [1, 2], as well as for different subtyping frameworks incorporating microenvironment-related and cancer cell-intrinsic signals [3, 4]. The current consensus framework (the consensus molecular subtypes, CMS) defines four biologically distinct subgroups with associations to clinicopathological factors (cancer stage and tumor localization), molecular markers (microsatellite instability [MSI] and *KRAS*/*BRAF*^V600E^ mutations), and patient survival [3]. CMS also reflect therapeutically relevant signaling pathways, such as enrichment with EGFR signaling in CMS2-epithelial/canonical tumors and angiogenic signals in the CMS4-mesenchymal/stromal group, suggesting that CMS could also be used for selection of standard targeted agents [5, 6]. However, retrospective analyses of randomized clinical trials comparing combination chemotherapies plus either anti-EGFR or anti-VEGF monoclonal antibodies in the first-line treatment of *KRAS* wild-type metastatic CRCs, showed inconsistent results with respect to the predictive value of CMS [7, 8]. These studies have highlighted the unsettled question of the suitability of the CMS framework in the metastatic setting [9].

CMS was originally developed for primary tumors, and metastatic lesions have different expression signals from the tumor microenvironment, as well as a different distribution of the clinicopathological and molecular features associated with CMS [10]. Furthermore, CMS4-mesenchymal/stromal tumors are associated with a poor prognosis in the primary setting [3, 6], while patients with CMS1-MSI/immune cancers have a particularly short survival after metastatic dissemination [7, 8, 11]. The CRC intrinsic subtypes (CRIS) were identified as a more uniform framework across different sources of CRC samples [4], but the clinical relevance of CRIS has not been equally well addressed. It has been suggested that also metastases can be grouped according to epithelial-like and mesenchymal-like expression signals [12, 13], but only few studies have sampled metastatic lesions.

The liver is the most common site of metastasis from CRC and approximately 30% of the patients develop liver metastasis, commonly with multiple lesions. This is associated with a 5-year overall survival (OS) rate of only approximately 15% [14], although liver resection offers a potential for long-term survival in a subset of the patients [15]. A few molecularly guided systemic treatment options have shown clinical benefit, including anti-EGFR agents in *KRAS*/*NRAS* (*RAS*) wild-type cancers with a left-sided primary tumor location [16], immune checkpoint inhibitors against MSI cancers [17, 18], and targeted combination therapies against *BRAF*^V600E^ mutated cancers [19]. Molecular pre-screening for therapy selection in the metastatic setting is most commonly based on the primary tumor, justified by the strong concordance between primary and metastatic tumors for the currently “actionable” genetic aberrations [20–22]. However, tumor heterogeneity is a major cause of treatment failure, illustrated by the clonal expansion of resistant subclones with acquired *RAS* mutations during anti-EGFR treatment [23]. Gene expression profiles are highly dynamic, and heterogeneity of CMS between matched primary tumors and metastases may be found in as many as 40% of patients [11], further highlighting the need for molecular profiling directly of metastatic lesions.

In this study we performed transcriptomic profiling and de novo subtype discovery and validation analyses of CRC liver metastases (CRLMs) from an in-house series and two external series of a total of 479 patients. The new subtyping framework was analyzed for biological characteristics by gene set enrichments, and for prognostic associations in relation to established transcriptomic frameworks (CMS and CRIS), clinicopathological factors, key genomic markers such as *RAS*, *BRAF*^V600E^ and *TP53* mutations, and intra-patient heterogeneity among metastatic lesions from 47 of the patients.

## Methods

### Patient material

A total of 829 samples from CRLMs, non-malignant liver tissue, primary CRCs, and pre-clinical CRC models have been analyzed in the study. The in-house series of metastatic CRC included 171 patients treated by hepatic resection at Oslo University Hospital between October 2013 and March 2018 (Table 1). The median age at surgery was 65 years (range 24–85) and the median follow-up time was 41 months. The patients had a median of 4 liver metastases (range 1–23) on radiological imaging before treatment, and fresh-frozen samples were collected from distinct metastatic lesions larger than 5 mm and from adjacent, macroscopically non-malignant tissue in the resected liver specimens. From these patients, 283 CRLM samples were analyzed. The dataset for intra-patient tumor heterogeneity analyses (totally 158 samples from 47 patients) included multiple metastatic lesions (from the same resection) from 42 patients (mean of 3 and median of 2 lesions per patient, range 2–7), 2–4 multiregional samples from each of 15 lesions, and 1–3 lesions from hepatic re-resection of 7 patients. Adjacent non-malignant liver tissue samples from 19 patients were also analyzed.

**Table 1:** Clinicopathological characteristics of patients with resected CRLM in the in-house series

Clinicopathological variable		Patients (total *n* = 171)	%
Gender, male		106	62
Primary tumor location			
	Proximal colon	36	21
	Distal colon	135	79
Primary tumor differentiation (unknown, *n* = 19)			
	Well	25	15
	Moderate	107	63
	Poor	20	12
Nodal status primary tumor (unknown, *n* = 8)			
	N0	50	29
	N1	62	36
	N2	51	30
Synchronous (within 6 months) liver metastasis		133	78
Previous resection/radiofrequency ablation of CRLM		39	23
Systemic oncological treatment prior to tumor sampling		156	91
	Neoadjuvant chemotherapy for this metastatic situation	131	77
	Previous chemotherapy before this metastatic situation	52	30
	Molecularly targeted treatment, previous or neoadjuvant	51	30
Radiofrequency ablation		22	13
R-status liver			
	R0-resection	71	42
	R1-resection^a^	91	53
	R2-resection^b^	9	5
Extra-hepatic disease		40	23
Multiple CRLM analyzed (patients, tumors, samples)		47/141/158	

^a^1 mm resectional margin or lesions treated with radiofrequency ablation

^b^ Macroscopic residual tumor in liver (visible at surgery or by radiological examination)

RNA and DNA were extracted using the Allprep DNA/RNA/miRNA Universal kit (Qiagen GmBH, Hilden, Germany), and nucleic acid concentrations were measured with Nanodrop spectrophotometry (Thermo Fisher Scientific, Waltham, MA, USA). RNA quality was assessed by the RNA integrity number (RIN) using the Bioanalyzer 2100 system (RNA 6000 Nano kit; Agilent Technologies, Santa Clara, CA, USA), and all samples had RIN > 6 (median 9.4).

Previously published in-house data from the primary tumor of 170 patients treated surgically for stage I–IV CRC at Oslo University Hospital [6], 34 CRC cell lines [24], and 15 patient-derived organoids (PDOs) grown from resected CRLMs [25] were included for comparison. Two publicly available gene expression datasets of resected CRLMs were downloaded from the Gene Expression Omnibus (GEO) under accession numbers GSE131418 (*n* = 141 CRLMs; MCC dataset [12]) and GSE73255 (*n* = 167 CRLMs [4]) and used for independent validation analyses.

An overview of the study materials and analyses is shown in Supplementary Figure 1 (Additional file 1: Fig. S1).

### Gene expression analyses

All CRLM samples (*n* = 283) and adjacent non-malignant liver tissue samples from 19 of the patients were analyzed for gene expression at exon-resolution on the GeneChip Human Transcriptome Array 2.0 (HTA 2.0; Thermo Fisher Scientific) using 100 ng of total RNA as input, and following the manufacturer’s protocol. The primary tumors and pre-clinical CRC models were analyzed on the same type of array in separate studies (GEO accession numbers GSE96528 [6], GSE79959 [26] and GSE97023 [24]). The in-house data represented two batches (primary CRCs and cell lines versus CRLMs, normal liver samples, and PDOs). The raw intensity CEL files were background corrected, normalized, summarized at the gene level, and log2 transformed using the robust multi-array average (RMA) method implemented in the justRMA function in the affy package [27] in R, using the custom Entrez CDF file (v22) from Brainarray [28]. Pre-processing was performed across sample types as defined by the downstream analyses (all 521 in-house samples; CRLMs and non-malignant liver tissue; or CRLMs only). Entrez IDs were converted to HGNC gene symbols using the org.Hs.eg.db package (v 3.7.0) from Bioconductor [29].

Principal component analysis (PCA) was performed in R by the prcomp function based on genes (*n* = 1000) with the highest standard deviation (SD) across samples, and hierarchical clustering was similarly performed using Manhattan distance and ward.D2 linkage in the R package stats. Differential gene expression analysis was performed by Empirical Bayes estimation in the R package limma [30], with Benjamini-Hochberg correction for the false discovery rate (FDR). Gene set enrichment analysis (GSEA) with FDR correction was performed using the camera function in limma on a collection of 57 CRC-related gene sets (Additional file 2: Table S3 and S4). Sample-wise liver scores were calculated by the gsva function implemented in the R package GSVA [31], based on a set of 157 genes with expression enrichment in the liver, retrieved from The Human Protein Atlas (https://www.proteinatlas.org/humanproteome/tissue/liver; version 18).

**Fig. 1:**
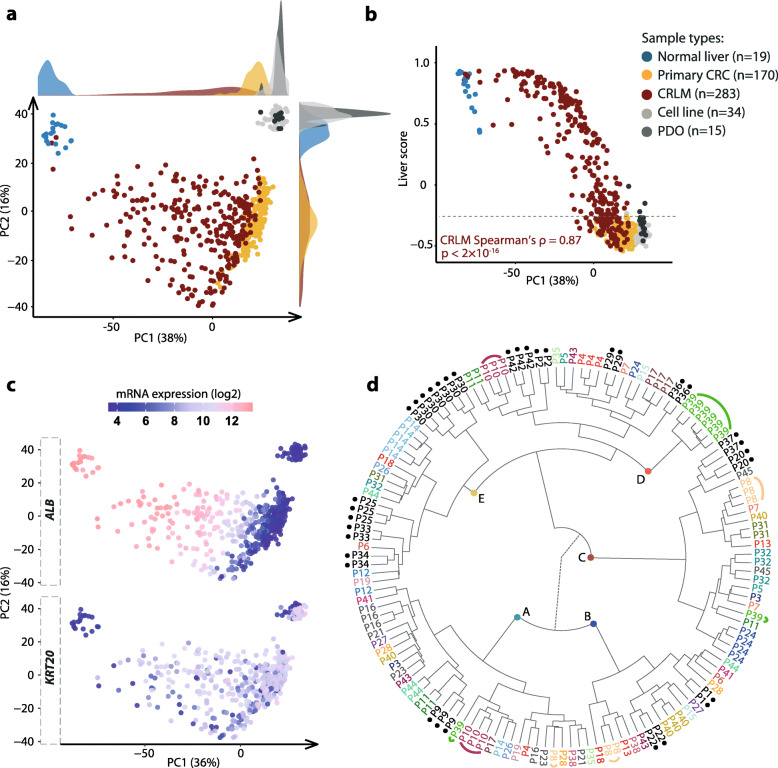
Comparison of gene expression profiles of CRLMs with normal liver tissue samples, primary CRCs, CRC cell lines, and PDOs. **a** PCA showed sample clustering based on sample type and tissue of origin. CRLMs had largest variation along PC1, as indicated by the density plot on top. **b** PC1 versus sample-wise liver scores calculated by GSVA of a set of genes highly expressed in the liver. The liver scores of CRLMs ranged from the normal liver tissue samples to the primary CRCs. 27% of the CRLMs had a liver score below the maximum score for primary CRCs, as indicated by the gray dashed line. **c** Repeated PCA plot of all samples along the PC1 and PC2 axes, colored according to the microarray expression levels of *ALB* and *KRT20*. The tree CRLM samples that clustered close to non-malignant liver samples in part **a** were excluded. **d** Hierarchical clustering of multiple (two to eight) distinct CRLMs from each of 45 patients. The tree is divided into five main branches, denoted A–E. Patients (*n* = 13) with adjacent clustering of all metastases are marked with black dots. Patients (*n* = 28) with separation of metastases into two or more main branches are represented by unique patient-wise colors. Patients (*n* = 4; P16, P21, P23, P45) with all metastases clustering within the same main branch, although not adjacent to each other, are colored gray. Three selected patients (P8, P10, P39) with separation of metastatic lesions on 2–3 of the main branches each are emphasized with arched lines

Analysis across the two different technical batches of the in-house gene expression data was performed in the initial exploratory step, presented as PCA in Fig. 1a–c. To evaluate the need for a batch correction, data were pre-processed in two batches and merged using the ComBat method in the R package sva [32]. PCA of batch-corrected data showed separation of pre-clinical models along the first principal component (PC1), as well as clustering of primary CRCs and CRLMs relative to normal liver samples (Additional file 1: Fig. S2). Furthermore, the sample-wise liver score and gene expression signal of the hepatocyte differentiation marker *ALB* were not significantly different between primary CRCs and CRLMs, inconsistent with the known and distinct gene expression patterns of liver tissue samples [33]. Accordingly, batch correction likely removed important biological distinctions between the sample types, and data were therefore presented without batch correction. However, we cannot conclude that the results shown in Fig. 1a–c are not impacted by more subtle technical variation between the two sample batches (particularly relevant for the comparison of primary CRCs and CRLMs).

### MSI and mutation analyses

MSI status was determined by PCR-based analysis of either the BAT25/26 mononucleotide markers or with the MSI Analysis System, version 1.2 (Promega, Madison, WI, USA). The CRLMs have previously been sequenced for hotspot mutations in *KRAS* and *NRAS* exons 2–4, *BRAF* exon 15, and for mutations in all coding regions of *TP53* (exon 2–11) [22].

### CMS and CRIS classification of CRLMs

One randomly selected CRLM sample from each patient in the in-house series was classified according to both the CMS and CRIS transcriptomic frameworks. For CMS classification, we have recently developed an algorithm tailored to CRLMs, taking into consideration the different distribution of clinicopathological factors and molecular subgroups in the primary and metastatic settings, as well as the different tumor microenvironment in the liver [34]. The tailored classifier is available in an updated version (v2.0.1) of the R package CMScaller (https://github.com/Lothelab/CMScaller). Using this classifier, 129 of the CRLMs (76%) were confidently classified. CRIS classification was performed using the cris_classifier function in the CRISclassifier R package [4] with default settings. This resulted in confident classification of 139 (82%) CRLMs.

### Unsupervised de novo transcriptomic classification of CRLMs

Unsupervised classification of CRLMs based on gene expression was performed using nonnegative matrix factorization (NMF) with the Brunet method, as implemented in the NMF R package [35, 36], with 100 repetitions and a pre-defined rank of 2 to 6. The classification was performed for single metastases from each patient to ensure sample independence (*n* = 169; the same tumor samples used for CMS and CRIS classification). Features for NMF included genes annotated as protein-coding, lincRNA and miRNA genes (*n* = 25969) in the BioMart database (retrieved April 2019) after a two-step filtering approach: (i) only genes that were upregulated in the CRLMs compared to the non-malignant liver tissue samples in unpaired differential expression analysis by limma were considered (*n* = 6247; log2 fold-change > 0, FDR-corrected *p* values ≤ 0.05); (ii) only genes with largest expression variation among the CRLMs (SD > 0.8; *n* = 514) were retained. The rationale for inclusion only of over-expressed genes in CRLMs was to reduce the influence of normal cell contamination in bulk tumor gene expression data (70% [110/157] of the liver-enriched genes retrieved from The Human Protein Atlas were among the 313 genes downregulated in CRLMs compared to non-malignant liver tissue). Gene expression values (log2-scale) were exponentially transformed (linear scale) prior to NMF. Classification at *K* = 5 was largely concordant when comparing thresholds for the input features of SD > 0.7 (*n* = 763 genes) and SD > 0.8 (Cohen’s *ĸ* = 0.87, 95% confidence interval [CI] = 0.84–0.94).

### LMS prediction model for classification of independent samples

A supervised random forest classifier for subtyping of independent samples into the five de novo LMS groups was trained on the CRLMs with a positive silhouette value in the initial LMS discovery analysis (*n* = 163 CRLMs, here called the training set). Template features were the same as the input for NMF. The best performing subset of genes (largest prediction accuracy) was identified by recursive feature elimination implemented with the rfe function in the R package caret [37], and with initial NMF class labels from the training set as outcomes. Function options were set to “parRF” method, 3 times 7-fold repeated cross-validation, “random” search for tuning parameter, “multiclass” summary function, and “Accuracy” metric. The weight of each gene included in the final model (*n* = 180, Additional file 2: Table S10) was calculated by the varImp function in the caret package. The trained model was then applied to all in-house CRLMs using the predict function in the R package stats. The performance of the prediction model was estimated for the training set used for LMS discovery (class labels from subtype discovery were considered “true”) using the confusionMatrix function in the caret package.

### Transcriptomic subtyping of external datasets

Two external gene expression datasets generated on Rosetta/Merck Human RSTA Custom Affymetrix 2.0 and Illumina HumanHT-12 V3.0 bead chips platforms were retrieved from GEO with accession numbers GSE131418 [12] and GSE73255 [4], respectively. For GSE131418, raw CEL files from 141 resected CRLMs in the MCC dataset were processed using the justRMA function in the affy package and the provided CDF file (HuRSTA_2a520709.cdf). Entrez IDs were mapped to HGNC symbols using the org.Hs.eg.db package and expression values for non-unique symbols were median aggregated. GSE73255 included 167 unique CRLMs retrieved using the getGEO function in the R package GEOquery. Probe IDs were converted to Entrez IDs and HGNC symbols using the illuminaHumanv4.db package from Bioconductor [29] and the org.Hs.eg.db package, respectively. Genes with the highest cross-sample variance were selected for entries with non-unique symbols, and expression values were log2 transformed. Both gene expression matrices were centered by the column/sample-wise mean and scaled by the column/sample-wise SD.

Supervised LMS prediction was performed on the two data sets separately, and according to the approach described for the independent in-house samples above, with the exception that new prediction models were trained with template features represented in each of the two external datasets (GSE131418: *n* = 480/514 genes, 93%; GSE73255: 462/514, 90%). In brief, a supervised random forest classifier was trained in the in-house training set (samples with known LMS labels), using recursive feature elimination to select the subset of the respective template gene sets with largest prediction accuracy (estimated by cross-validation in the training set; GSE131418: *n* = 230 genes in the final model; GSE73255: *n* = 390). The trained models were applied to the corresponding external dataset using the predict function in R.

For comparison with the LMS predictions, unsupervised de novo transcriptomic subtyping was also performed by NMF of each of the two external datasets following the same approach as for the in-house series, and using the set of overlapping template genes on each platform (GSE131418: *n* = 480 genes; GSE73255: *n* = 462).

### LMS1 mini-classifier

A two-class random forest model (LMS1 versus rest of the subtypes) was trained using the train function in the caret package on differentially expressed genes identified from limma analysis comparing LMS1 to all other subtypes in the in-house training series (FDR-corrected *p* ≤ 0.05, log fold-change ≥ 1.6, *n* = 9 genes). The prediction model was trained using 7-fold leave-one-out cross-validation. The optimal value of the mtry parameter was identified using the tuneLength option in the train function. Class labels were predicted using the predict function and were compared with original class labels in the complete in-house dataset.

For assessment of this 9-gene LMS1 mini-classifier in the external datasets, CRLM samples from the in-house training series, GSE131418, and GSE73255 were merged based on common genes and batch corrected using the ComBat function in the R package sva. Missing values for *UCA1* in GSE73255 were imputed by its median expression across the batch-corrected dataset. The random forest model for the 9-gene signature was re-trained on the batch-corrected in-house training series and applied to the full dataset using the predict function. The prediction accuracy of the trained model (LMS1 versus LMS2-5 distinction) was 100% for re-classification of the in-house training series.

### Statistical analysis

All statistical tests were two-sided and performed in R (v3.5). Fisher’s exact, Pearson’s chi-squared, *t*-test, and Wilcoxon tests were performed using fisher.test, chisq.test, t.test, and wilcox.test functions in R package stats, respectively. Spearman’s correlation was calculated using stat_cor function in R package ggpubr. Cohen’s kappa was calculated using the confusionMatrix function in R package caret. Five-year OS and cancer-specific survival (CSS) curves were estimated with the Kaplan-Meier method using the survfit function in the R package survival. Pairwise log-rank tests were performed to compare survival curves using the pairwise_survdiff function in the R package survminer, with the method for *p* value adjustment set to the Benjamini-Hochberg procedure. The time to event or censoring was calculated from initiation of treatment for the CRLMs, either neoadjuvant treatment or hepatic resection. All deaths were registered as events for OS, and death from CRC was defined as an event for CSS, with censoring of patients who died from other causes. Patients without events the first 5 years of follow-up were censored. Hazard ratios (HR) were calculated in univariable and multivariable Cox proportional hazards analyses using the coxph function in R package survival and *p* values were calculated using Wald test. The proportional hazards assumption was checked using the cox.zph function and was supported for all Cox models.

## Results

### Variable impact of the tumor microenvironment on gene expression profiles of CRC liver metastases

To assess the primary-to-metastasis transcriptomic landscape in CRC, gene expression profiles of 283 resected liver metastasis samples and 19 non-malignant liver tissue samples from 171 patients (Table 1) were initially compared with primary CRCs (*n* = 170) and pre-clinical CRC models derived from primary tumors (*n* = 34 cell lines) or resected CRLMs (*n* = 15 PDOs). In PCA, the CRLMs ranged from the primary CRCs to the non-malignant liver samples along PC1, although the closer vicinity to the primary CRCs indicated resemblance of the metastasized cancer cells to the tumors of origin (Fig. 1a). The CRLMs had a larger spread along PC1 than the primary CRCs (10–90th percentile range of PC1 values of 29 and 6.3, respectively), indicating a highly variable degree of influence from the liver tumor microenvironment in CRLMs. This was confirmed by calculation of a sample-wise liver score based on genes with high expression in the liver (see “Methods”), which correlated strongly with PC1 of the CRLMs (Fig. 1b). The liver scores of the CRLMs spanned from the non-malignant liver samples (range 0.40 to 0.89) to the primary CRCs (range − 0.3 to − 0.58) and cell lines (range − 0.27 to − 0.54). The large variation in the degree of tumor microenvironment infiltration in the CRLMs was further illustrated by the gene expression levels of the hepatocyte differentiation marker *ALB*, which was highest in the non-malignant liver samples and decreased gradually in the CRLMs along PC1 (Fig. 1c). The opposite expression pattern was found for the intestinal differentiation marker *KRT20*. Notably, 27% of the CRLMs (75/283) had liver scores within the range of the primary CRCs (liver score < − 0.3; Fig. 1b), suggesting negligible influence from the liver tumor microenvironment in these samples. Three CRLM samples from three patients clustered close to the non-malignant liver samples in PCA (Fig. 1a) and were excluded from further analyses.

The majority of patients had received chemotherapy prior to sampling of the CRLMs (Table 1). PC1 values were slightly lower for CRLMs treated in a neoadjuvant setting compared to chemo-naive and/or previously treated tumors (one randomly selected sample per patient; Additional file 1: Fig. S3), indicating an impact of neoadjuvant chemotherapy on the gene expression profiles. Among other clinicopathological characteristics, only R2 resections in the liver and extra-hepatic disease were associated with PC1 values and the liver scores (Additional file 2: Table S1).

Exploratory analyses indicated pronounced intra-patient transcriptomic heterogeneity among metastatic lesions, illustrated by hierarchical clustering of 2–8 CRLMs from each of 45 patients (total *n* = 139 lesions; Fig. 1d). Only 13 patients (29%) had multiple CRLMs that clustered together, while 62% of the patients (28/45) had CRLMs that separated on at least two of the five main branches. The remaining 9% of the patients (4/45) had metastases that clustered on the same main branch, although not adjacent to each other. Patient-wise clustering versus separation of samples was not associated with exposure to neoadjuvant chemotherapy (Fisher’s exact *p* = 0.3). However, a comparison of CRLM liver scores showed that hepatocyte infiltration was higher in samples from patients with separation of metastases into different clusters, compared to patients with adjacent sample clustering, indicating an association with inter-metastatic heterogeneity (although not statistically significant; Wilcoxon *p* = 0.07).

### De novo transcriptomic subtypes of CRLM

**Fig. 2:**
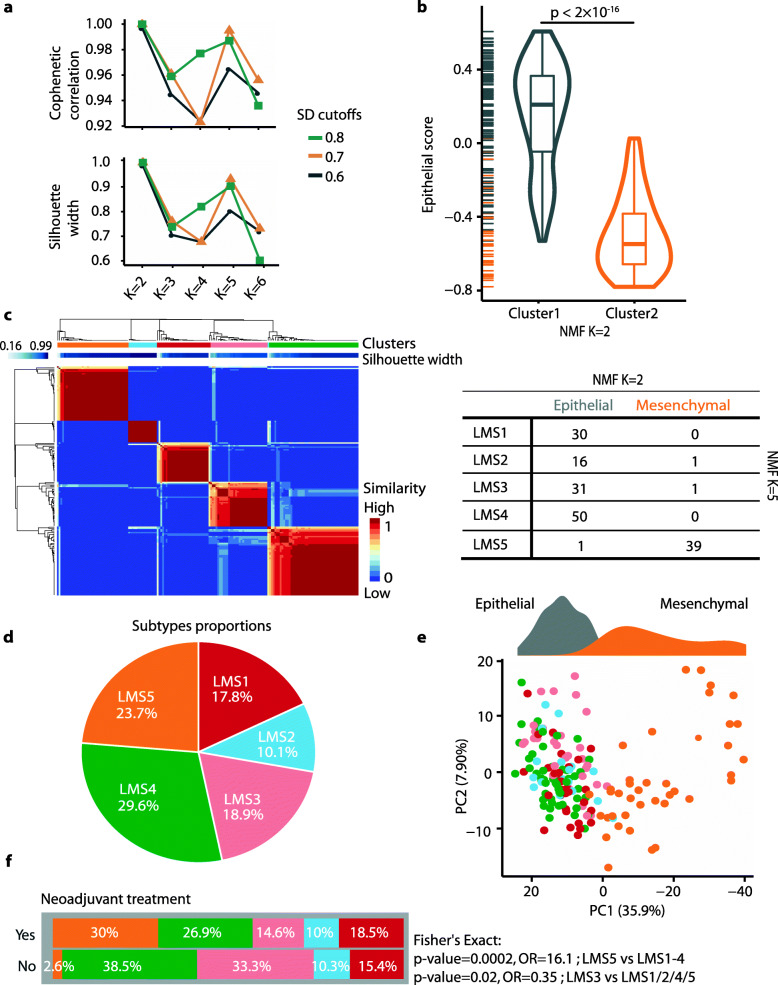
Unsupervised de novo subtyping of CRLMs based on gene expression. **a** Quality metrics from NMF classification using input gene sets defined by three different thresholds for the cross-sample SD indicated that the optimal number of sample clusters (K) was either 2 or 5. **b** The sample clusters at *K* = 2 factorization were most strongly separated by epithelial-mesenchymal characteristics, as illustrated with a sample-wise epithelial score calculated by GSVA (*p* value from *t*-test). **c** Heatmap of NMF clustering output at *K* = 5 factorization. The top annotation bars indicate sample clusters and the sample-wise silhouette width in each cluster. The red-blue color intensity in the heatmap represents the within-cluster similarity of each sample. Cross-tabulation of samples at *K* = 2 and *K* = 5 factorizations indicates that the mesenchymal subtype from *K* = 2 is largely retained also at *K* = 5. **d** Pie chart showing the proportion of samples in each of the de novo liver metastasis subtypes (LMS1-5) at *K* = 5. **e** PCA plot of samples based on the input gene set for NMF (cross-sample SD > 0.8) and colored according to LMS group, confirms strong separation of the mesenchymal subtype (LMS5) from the four epithelial subtypes (LMS1-4) along PC1. The density plot on the top shows the distinction between the epithelial and mesenchymal sample clusters from *K* = 2 factorization. **f** The proportion of LMS5 samples was higher among CRLMs exposed to neoadjuvant chemotherapy, but there was no significant difference between treatment groups for LMS1, LMS2, and LMS4

By adapting CMS classification to liver metastases and developing a new version of the R package CMScaller [38] (v2.0.1), we have shown that CMS has limited discriminatory power in CRLM [34]. Most metastatic lesions were classified into one of only two subtypes, based on epithelial-mesenchymal characteristics (Additional file 1: Fig. S4). In addition, CMS classification was strongly influenced by systemic treatment prior to sampling, showing strong enrichment with CMS4-mesenchymal/stromal tumors and concomitant depletion of CMS2-epithelial/canonical among tumors exposed to neoadjuvant chemotherapy (Additional file 1: Fig. S4). We therefore investigated the potential to develop a new intrinsic classification framework for CRLM that captures additional biological information. Unsupervised classification of single CRLMs from each patient (*n* = 169 samples, patient-wise random selection) was performed by NMF of a filtered set of 514 genes (Additional file 2: Table S2), selected both for having upregulated expression in CRLMs compared to non-malignant liver tissue samples, and for high expression variation among the CRLMs (see “Methods”). Quality metrics from NMF classification, including the cophenetic correlation coefficient and silhouette width, were highest at *K* = 2 and *K* = 5 across different input gene sets defined by the expression variation threshold (Fig. 2a). GSEA of a custom collection of CRC-related gene sets (*n* = 57; Additional file 2: Table S3, Additional file 1: Fig. S5) indicated that sample classification at *K* = 2 resulted in subtypes with predominantly epithelial (cluster 1: 76% of tumors) or mesenchymal (cluster 2: 24% of tumors) characteristics (Fig. 2b). Classification at *K* = 5 resulted in four additional sub-classes within the epithelial subtype, with a 98% concordance between epithelial and mesenchymal-like subtypes at the two factorization levels (Cohen’s *ĸ* = 0.98, 95% CI = 0.95–1; Fig. 2c).

The five de novo sample clusters, hereafter called liver metastasis subtypes (LMS), each represented 18% (LMS1), 10% (LMS2), 19% (LMS3), 30% (LMS4), and 24% (LMS5) of the tumors (Fig. 2d, Additional file 1: Fig. S6a-b). PCA confirmed that epithelial (LMS1-4) versus mesenchymal (LMS5) characteristics represented the primary distinction of samples along PC1 (Fig. 2e). There was little difference in the distribution of liver scores among the subtypes, indicating that the LMS framework was not confounded by hepatocyte infiltration (Additional file 1: Fig. S6c). However, LMS5-mesenchymal was significantly enriched among CRLMs exposed to neoadjuvant chemotherapy (Fig. 2f). Among the four epithelial subtypes, only LMS3 was significantly depleted in the chemotherapy-exposed group.

### Enrichments with specific cell types and *RAS*/*TP53* co-mutations in the LMS framework

**Fig. 3:**
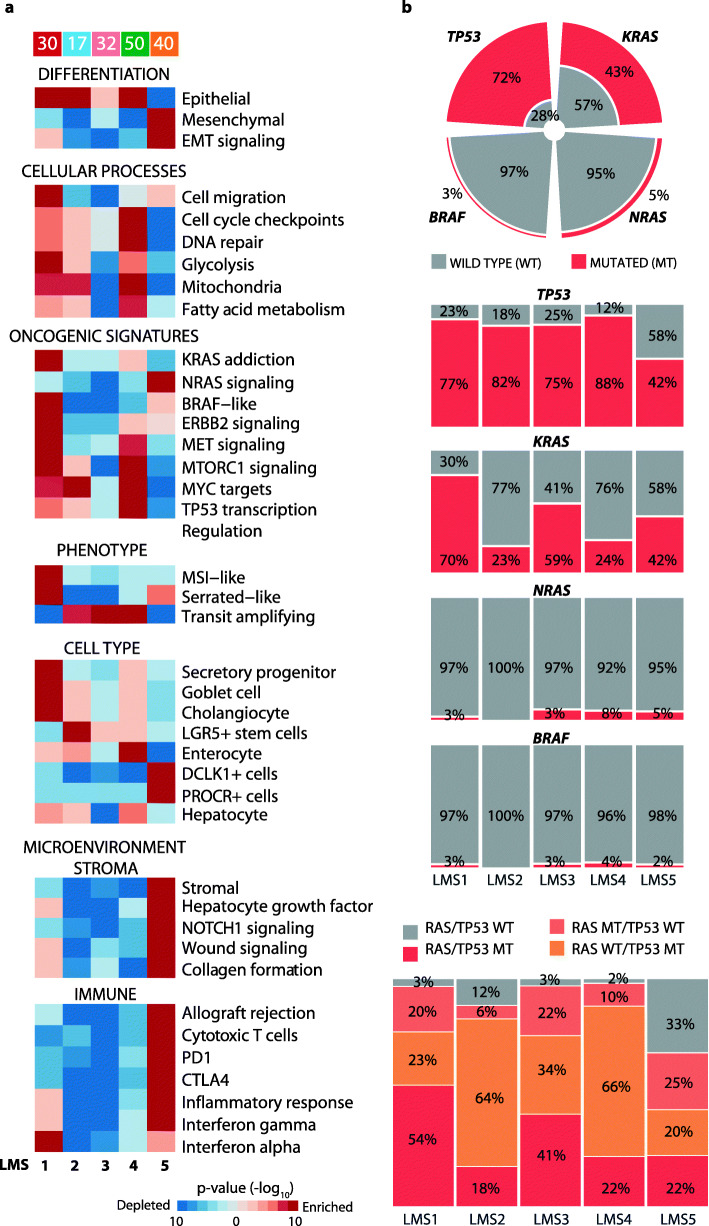
Molecular characteristics of the de novo LMS framework. **a** GSEA of selected gene expression signatures shows distinct patterns of activated (red) or downregulated (blue) pathways. The color intensities represent *p* values from comparison of each subtype against all others (analyzing one randomly selected CRLM sample from each patient, *n* = 169). **b** From top: *TP53/KRAS/NRAS/BRAF*^V600E^ mutation frequency across patients, with and without subtype stratification (for the latter, calculated per subtype). Bottom: Frequency of *RAS*/*TP53* co-mutations in each subtype

Distinct patterns of biological processes among the LMS groups were found by GSEA (Fig. 3a, Additional file 2: Table S4). LMS5-mesenchymal CRLMs were enriched with tumor microenvironment signals, including a strong stromal component and a high relative expression of immune-related gene signatures. LMS1 had strong gene expression-based MSI characteristics and included the single MSI-high CRLM (CRLMs from all other patients [168/169] were confirmed MSS). The MSI-high sample had the third highest MSI-like score, and most MSS tumors in LMS1 had stronger MSI-like characteristics than MSS tumors in LMS2-5 (Additional file 1: Fig. S7a). Notably, the MSI-like score had only a weak correlation with cytotoxic T cell signals among the CRLMs (Spearman’s *ρ* = 0.2; Additional file 1: Fig. S7b), consistent with the predominantly weak immune response signals in LMS1. LMS1 was further characterized by several oncogenic signatures in the MAPK and MET signaling pathways (including KRAS and BRAF signatures), as well as cancer aggressiveness (cell migration, hypoxia) and a signature of resistance to the standard chemotherapeutic agent 5-fluorouracil. LMS2-4 all had a transit amplifying-like phenotype. LMS2-3 showed enrichments with few other signaling pathways, while LMS4 presented with strong metabolic signals (partly shared with LMS1 and LMS2), TP53 transcriptional activity, and cell cycle-associated signatures (cell cycle checkpoints and DNA repair mechanism; Fig. 3a).

Cell type-specific gene markers extracted from published single-cell RNA sequencing studies (Additional file 2: Table S4) indicated clear differences in the most dominating cell type of origin of each subtype. LMS1 CRLMs were highly enriched with genes related to secretory progenitor cells, mucus-secreting goblet cells (for example, *MUC2* and *MUC4*), and liver cholangiocytes (for example, *KRT7*, *KRT19*, *EPCAM*, *SOX9*). LMS2 strongly expressed core gene markers of LGR5+ intestinal stem cells (*LGR5*, *OLFM4, ASCL2, SMOC1*, and *MSI1*). No inference of the cell type of origin could be made for LMS3 CRLMs. LMS4 showed marked expression of absorptive enterocyte markers, and LMS5-mesenchymal tumors showed strong expression of markers of quiescent stem cells (*DLCK1+*, *PROCR+*).

The five LMS groups were further analyzed for potential enrichment with key genomic markers of CRC beyond MSI status, including mutations of *TP53*, *KRAS, NRAS*, and *BRAF*^V600E^ (Fig. 3b). *TP53* mutations were common across all subtypes, but with a significantly lower mutation frequency in LMS5-mesenchymal tumors (LMS5 versus LMS1-4: Fisher’s exact *p* = 5 × 10^−6^, odds ratio [OR] = 0.2; Additional file 2: Table S5). *KRAS* mutations were enriched in LMS1-secretory/MSI-like tumors (LMS1 versus LMS2-5: *p* = 0.002, OR = 3.9), although the mutation frequency was not significantly higher in LMS1 than LMS3 separately (*p* = 0.4). Notably, there was enrichment with the gene expression-based KRAS addiction signature in LMS1 also when analyzing *KRAS* mutated CRLMs only, further supporting preferential KRAS signaling in LMS1 (Additional file 1: Fig. S7c). *NRAS* and *BRAF*^V600E^ had low mutation frequencies in all subtypes, and there were no significant enrichments. *RAS* and *TP53* were co-mutated in 31% (52/169) of the patients, and the co-mutations were enriched in LMS1-secretory/MSI-like CRLMs (LMS1 versus LMS2-5: *p* = 0.005, OR = 3.2; no significant difference between LMS1 and LMS3 separately: *p* = 0.4; Fig. 3b). GSEA of *RAS/TP53* co-mutated tumors only showed similar results to the analyses across all tumors, supporting enrichment with several oncogenic signatures in the MAPK and MET signaling pathways in LMS1 (Additional file 1: Fig. S8). TP53 transcriptional activity was enriched in LMS1 and LMS4, and LMS4 (together with LMS2) showed significant enrichment with *TP53* mutations in a *RAS* wild-type background (LMS2/4 versus LMS1/3/5: *p* = 8 × 10^−4^, OR = 3.8, 95% CI = 1.6–9.7).

### Poor prognosis associated with LMS1-secretory/MSI-like CRLMs

**Fig. 4:**
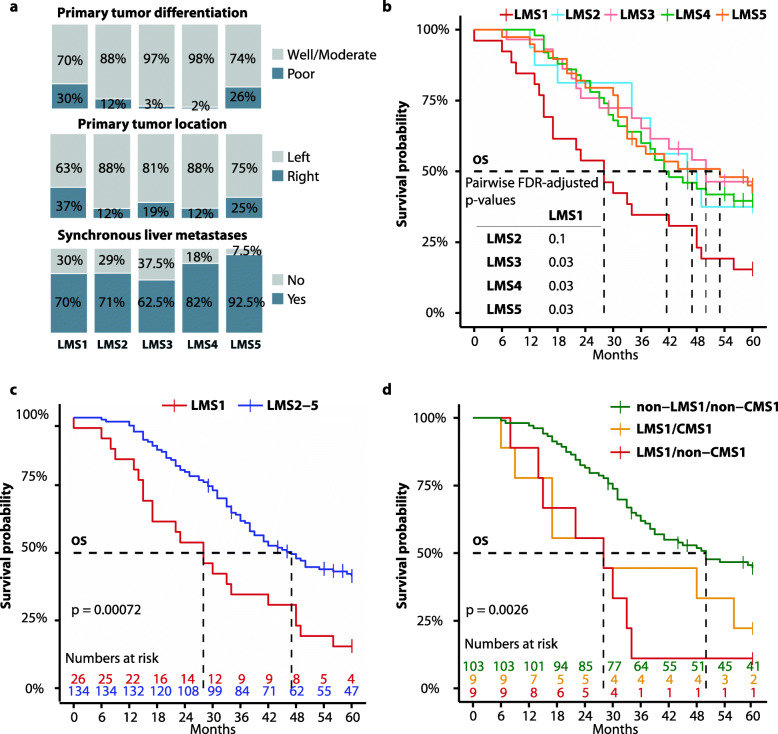
Associations of LMS with clinicopathological factors and patient outcome. **a** Subtype-wise frequency of clinicopathological variables with a significantly different distribution across the subtypes. Kaplan-Meier plots of 5-year OS stratified **b** according to the individual LMS groups, **c** by LMS1 versus LMS2-5 combined and **d** in combination with translated CMS subtypes as indicated. *P* values are calculated by log-rank test and in **b** FDR corrected by the Benjamini-Hochberg procedure

Several clinicopathological variables were differently distributed across the LMS groups (Additional file 2: Tables S5-S6). LMS1-secretory/MSI-like and LMS5-mesenchymal were enriched with CRLMs originating from poorly differentiated and proximal (right-sided) primary tumors compared to LMS2-4 (tumor differentiation: OR = 8.4, 95% CI = 2.5–36.4, *p* = 9 × 10^−5^; tumor location: OR = 2.6, 95% CI = 1.1–6.0, *p* = 0.02; Fisher’s exact test; Fig. 4a). Synchronous liver metastases were most frequently found in the LMS5-mesenchymal group (OR = 4.6, 95% CI = 1.3–24.6, *p* = 0.009). Furthermore, analyses of the 160 patients with R0/R1 resections in the liver showed prognostic associations to 5-year OS and CSS. Patients in the LMS1-secretory/MSI-like group had a 5-year OS rate of 15%, which was lower than for patients with LMS2-5 CRLMs, analyzed both individually (significantly different for each of LMS3-5; Fig. 4b) and collectively (HR = 2.2, 95% CI = 1.4–3.6, Wald test *p* = 9 × 10^−4^; Fig. 4c). A similar association was found with 5-year CSS as the endpoint (LMS1 versus LMS2-5: HR = 1.9, 95% CI = 1.2–3.3, Wald test *p* = 0.01; Additional file 1: Fig. S9). Notably, patient stratification based on epithelial or mesenchymal characteristics (from NMF classification at *K* = 2) had no prognostic associations (Additional file 1: Fig. S10). Multivariable Cox proportional hazards analyses including the clinicopathological parameters with univariable prognostic associations (patient gender, primary tumor differentiation grade, systemic oncological treatment prior to tumor sampling, R2 resection in the liver, and extra-hepatic disease; Additional file 2: Table S7) showed that the LMS framework (LMS1 versus LMS2-5) was an independent prognostic factor for both 5-year OS and CSS (adjusted HR = 2.4, 95% CI = 1.4–4.0, Wald test *p* = 1 × 10^−4^, and adjusted HR = 2.1, 95% CI = 1.2–3.7, Wald test *p* = 0.008, respectively, Additional file 2: Table S8). Furthermore, exclusion of patients with extra-hepatic disease and/or R2 resections in the liver (the clinicopathological factors with the strongest prognostic association) did not preclude the prognostic value of the LMS1-secretory/MSI-like group (Additional file 1: Fig. S11a).

*RAS*/*TP53* co-mutations were also associated with a worse 5-year OS (HR = 1.6, 95% CI = 1.1–2.5, Wald test *p* = 0.02 among patients with R0/R1 resection in the liver), and to investigate whether enrichment with co-mutations was the underlying factor for the prognostic value of LMS1-secretory/MSI-like CRLMs, we compared patients with LMS1 and LMS2-5 tumors with/without co-mutations. This indicated that LMS1 was associated with a poor patient survival independent of co-mutation status in a bivariable analysis (adjusted HR = 2.0, 95% CI = 1.2–3.3, Wald test *p* = 0.004), with worst prognosis for co-mutated LMS1, but no significant difference between co-mutated LMS2-5 and LMS1 without co-mutations (Additional file 1: Fig. S11b).

### Comparison of LMS with established transcriptomic frameworks

A direct comparison of LMS with the CMS (adapted to the liver metastatic setting) and CRIS frameworks showed only moderate subtype concordances, although LMS did not represent a statistically independent subtype distribution (Table 2). Notably, only 66% of CMS4-mesenchymal CRLMs (38/58) were included in LMS5-mesenchymal. Furthermore, 91% (10/11) of CMS1-MSI/immune CRLMs were found in LMS1, but CMS1 constituted only 45% (10/22) of the total number of tumors in this de novo subtype. Combined survival analyses of the LMS and CMS frameworks in patients with R0/R1 resections in the liver, focusing on the poor-prognostic subtypes LMS1 and CMS1, indicated that LMS provided the strongest prognostic stratification. The worst prognosis was found for patients classified as LMS1/non-CMS1 (5-year OS rate of 11%), followed by LMS1/CMS1 (22%) and non-LMS1/non-CMS1 (45%), respectively (log-rank *p* ≤ 0.004 for both 5-year OS and CSS; Fig. 4d).

**Table 2:** Correspondence of the de novo subtypes with CMS and CRIS in resected CRLMs

		Translated CMS				CRIS				
		CMS1	CMS2	CMS3	CMS4	CRIS-A	CRIS-B	CRIS-C	CRIS-D	CRIS E
De novo subtypes	LMS1	10	4	1	7	13	12	1	0	3
	LMS2	0	8	0	1	4	1	6	1	2
	LMS3	0	15	0	10	3	3	4	10	5
	LMS4	0	32	0	2	4	3	31	2	5
	LMS5	1	0	0	38	9	3	4	8	2
		*χ*^2^ = 151.9, *p* < 2 × 10^−16^				*χ*^2^ = 82.9, *p* = 4 × 10^−11^				

With respect to the LMS and CRIS (Additional file 1: Fig. S12) frameworks, the best subtype concordance was found between CRIS-C and LMS4 (69% [31/45] of samples in LMS4 were also CRIS-C), while 86% of samples in LMS1 were either CRIS-A or CRIS-B (Table 2). Survival analysis focused on LMS1 and CRIS-B showed a survival rank with worst outcome for LMS1/CRIS-B > LMS1/non-CRIS-B > non-LMS1/CRIS-B > non-LMS1/non-CRIS-B (log-rank *p* < 0.006 for both OS and CSS; Additional file 1: Fig. S12e).

### LMS1-secretory/MSI-like and LMS5-mesenchymal define distinct subtypes of CRLMs across independent datasets

**Fig. 5:**
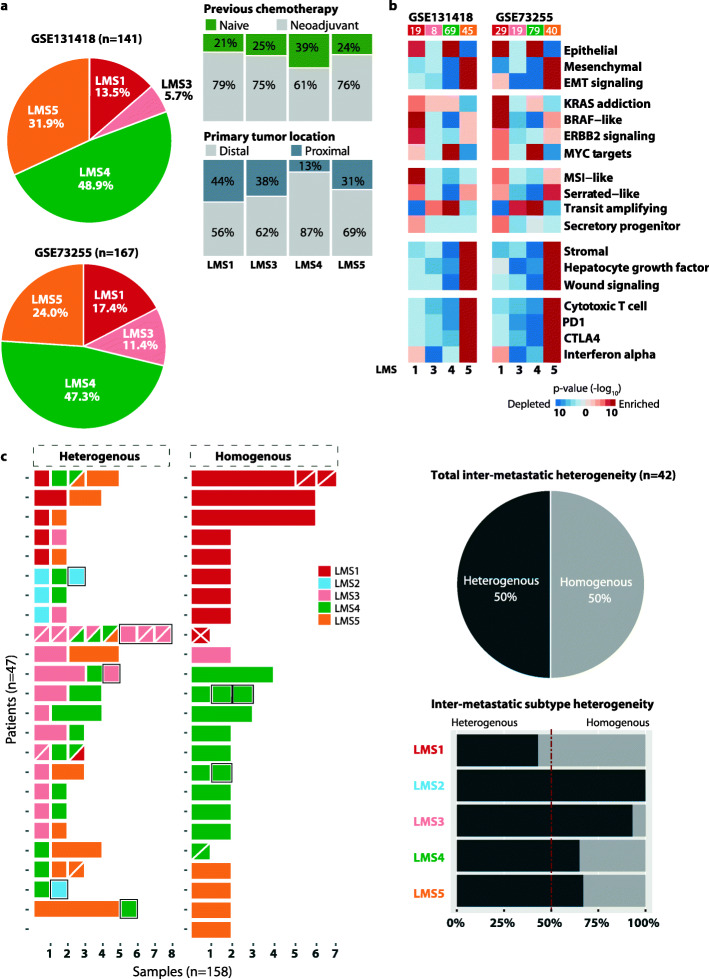
LMS predictions and intra-patient heterogeneity in additional CRLM samples. **a** Subtype distributions from LMS predictions in two publicly available datasets of resected CRLMs, also according to available clinical information. **b** GSEA results for selected signatures in each external series corresponded fairly well with the patterns observed in the in-house series. **c** Left panel: LMS predictions of multiple CRLM samples from a subset of patients in the in-house series. Each horizontal bar represents a patient, categorized according to heterogeneous or homogenous LMS classifications, and the length of the bars corresponds to the number of samples analyzed. Multiregional samples analyzed from each of 15 metastatic lesions are separated by white diagonal lines. Samples/lesions from repeated hepatic resections of seven patients are indicated with a black outline, including one patient with three resections. Right panel: the pie chart summarizes the proportion of overall intra-patient inter-metastatic subtype heterogeneity among the 42 patients with multiple metastatic lesions from the same hepatic resection. The bar plot below shows the proportion per LMS group and is calculated patient-wise among all patients who have at least one metastatic lesion/sample (from the same resection) classified in the specified subtype

To investigate the LMS framework in independent samples, a random forest LMS prediction model was developed (see “Methods”) and initially applied to two external gene expression datasets of 141 (GSE131418) and 167 (GSE73255) resected CRLMs analyzed on two separate microarray platforms [4, 12]. In comparison with the in-house dataset, there was a skewed distribution of LMS2-4 in both external datasets (Fig. 5a). The LMS4 group encompassed a relatively large proportion of samples, at the apparent cost of samples classified as LMS2 (missing from both datasets) or LMS3. The remaining subtype distributions were largely proportional to the in-house material, and GSEA indicated that several LMS characteristics were recapitulated in both independent datasets (Fig. 5b, Additional file 1: Fig. S13). LMS1 was found to have an epithelial and secretory phenotype with strong MSI-like and BRAF-like expression signals. LMS3 and LMS4 both had a transit amplifying phenotype, and LMS4 tumors additionally had strong signaling of MYC targets. LMS5 was identified as the only mesenchymal-like subtype and presented with a strong stromal and immune component. Investigation of the available clinicopathological information (in the GSE131418 dataset) supported that the subtype distribution was not associated with exposure to neoadjuvant treatment (Fisher’s exact *p* = 0.3) and that LMS1 CRLMs were more likely to originate from proximal primary tumors (OR = 2.9, 95% CI = 0.9–9.2, *p* = 0.04; Additional file 2: Table S9, Fig. 5a).

Notably, de novo transcriptomic subtyping by unsupervised NMF of each of the two external datasets supported that the optimal number of sample clusters was four. Matching of de novo subtypes with LMS predictions (based on the NMF sample cluster with the largest number of sample overlaps) also supported a larger relative proportion of LMS4 samples compared to the in-house series, at the apparent cost of LMS2 in particular (Additional file 1: Fig. S14a). Comparisons of the sample-wise posterior probabilities from the LMS prediction model (for LMS1, LMS3, LMS4, and LMS5) with cluster membership probabilities for the related NMF sample cluster showed a significant positive correlation for each subtype in each dataset, indicating correspondence between LMS predictions and unsupervised classification (Additional file 2: Fig. S14b). However, the correlations varied in strength and were strongest for LMS5 (Spearman’s *ρ* ≥ 0.85, *p* < 2 × 10^−16^) and weakest for LMS3 (Spearman’s *ρ* ≥ 0.23, *p* ≤ 0.005) in both datasets.

### Frequent intra-patient inter-metastatic subtype heterogeneity does not confound the prognostic value of LMS1

The random forest LMS prediction model was also applied to multiple additional CRLM samples from each of 47 patients in the in-house series (total *n* = 158 samples) to analyze intra-patient tumor heterogeneity. The prediction model had an overall balanced classification accuracy of 98% among the samples also included for initial subtype discovery (95% CI = 94-99, Additional file 2: Table S10). Intra-patient inter-metastatic subtype heterogeneity was observed in 21 (50%) of the 42 patients with multiple distinct lesions from the same hepatic resection, and intra-tumor heterogeneity was observed in 5 (33%) of the 15 lesions with multiregional samples (Fig. 5c). LMS1 was the least heterogeneous subtype, with inter-metastatic heterogeneity in 43% of the patients (6 of 14) with at least one LMS1 CRLM/sample, while LMS2 and LMS3 were most heterogeneous (in 100% and 93% of the patients, respectively).

Intra-patient inter-metastatic subtype heterogeneity was not associated with patient survival (log-rank *p* > 0.2 for 5-year OS and CSS; Additional file 1: Fig. S15a). However, considering the high frequency of subtype heterogeneity, we investigated its possible influence on the prognostic associations of LMS1. There was no statistical survival difference between patients with homogeneous LMS1 CRLMs and patients with inter-metastatic LMS1 heterogeneity, although these analyses were based on a small number of patients (Additional file 1: Fig. S15b). We further analyzed the impact of LMS1 heterogeneity in the complete patient series (*n* = 160 patients with R0/R1 resections of the liver) by switching the inclusion of patients with inter-metastatic LMS1 heterogeneity between the LMS1 group and the LMS2-5 group. This indicated no impact of tumor sampling or tumor subtype heterogeneity on the prognostic value of LMS1 (Additional file 1: Fig. S15c-d).

### Development of LMS1 mini-classifier

We explored the potential to identify the clinically relevant subgroup of LMS1 CRLMs using a simpler test based on a small number of genes. A two-class mini-classifier containing genes with high relative expression in the LMS1 group (*n* = 9 genes; *GCNT3*, *CTSE*, *REG4*, *TCN1*, *LCN2*, *DSG3*, *UCA1*, *SERPINB5*, and *MUC17*) was constructed in the in-house training series (single samples from each of the 169 patients). When applied to the complete in-house set of 280 CRLM samples, the classifier provided largely concordant classifications (LMS1 versus LMS2-5; Cohen’s *k* = 0.86). Single-sample gene set scores calculated by GSVA [31] of the nine LMS1 mini-classifier genes correlated strongly with single-sample scores for gene signatures enriched in LMS1 tumors, including the secretory progenitor signature, the MSI-like signature, and the KRAS addiction score (Spearman’s *ρ* > 0.6 across samples, *p* < 2 × 10^−16^; Additional file 1: Fig. S16). Furthermore, the mini-classifier accurately captured the prognostic association of the LMS1 group (5-year OS: HR = 2.2, 95% CI = 1.4–3.6, Wald test *p* = 0.001; Additional file 1: Fig. S17), suggesting that this 9-gene mini-classifier can be used for independent prognostic stratification of resected CRLMs. Application to the two external datasets (GSE131418 and GSE73255 combined) showed a prediction accuracy of 0.89 (95% CI 0.85–0.92) for the LMS1 versus LMS2-5 distinction.

## Discussion

**Fig. 6:**
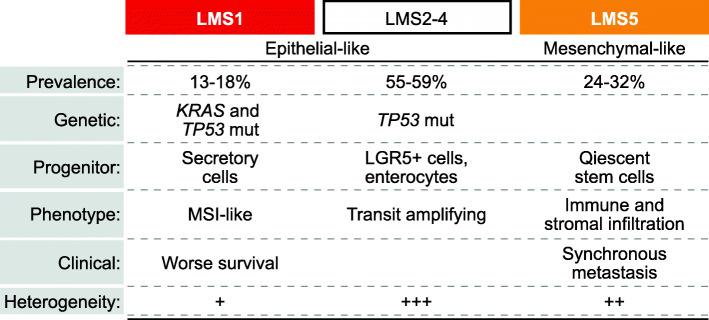
Overview of the de novo liver metastasis subtypes. The main characteristics of each subtype are summarized. Mut, mutations

The current consensus framework for transcriptomic subtyping of CRC [3] is widely adopted for primary tumors, but data supporting the need for a metastasis-oriented framework are growing. This study presents a new framework dedicated to liver metastases, summarized in Fig. 6. LMS showed only a moderate level of classification concordance with CMS, despite comparison with CMS classes obtained from a tailored classification of resected CRLM tissue samples [34]. LMS provided stronger biological and prognostic discriminatory power than CMS in this setting. Similar results were obtained by a comparison between LMS and CRIS classification [4], although CRIS by design should be less influenced by the CRC sample source. This highlights context-dependency of transcriptomic classifications of CRCs and supports an added value from analyses of metastatic lesions. We confirm the primary distinction between epithelial-like and mesenchymal-like tumors that has been shown also in previous studies focused on metastatic CRC [4, 12], including enrichment with MYC and cell cycle signals in the former group, as well as strong tumor microenvironment signals (both immune and stromal) in the latter. However, LMS showed a potential for further sub-stratification of epithelial-like CRLMs, and this uncovered the poor-prognostic LMS1 group. LMS1 clearly had distinct biological characteristics and was faithfully identified with a prevalence of 13–18% by subtype prediction across different external CRLM sample series and analysis platforms. Notably, the distinction among the remaining epithelial-like groups, LMS2-4, was less clear and not well reproduced. The main added value of the LMS framework therefore appeared to be the recognition of LMS1, and we therefore constructed an LMS1 mini-classifier based on nine genes with subtype-enriched expression, to facilitate analyses in additional CRLM sample series.

The clinically relevant and poor-prognostic LMS1 CRLMs had gene expression features associated with secretory progenitor cells and an MSI-like phenotype. This strengthens the current data suggesting an aggressive biology of metastasizing MSI-high CRCs, in contrast to the survival benefit associated with MSI in the primary setting [39]. Patients with MSI-high metastatic cancers have a clinical benefit with immune checkpoint inhibitors, likely associated with the high tumor mutational burden and strong anti-tumor immune responses. However, very few resectable CRLMs are MSI-high [40], and MSI-high status was found in only one of the patients in this series (< 1%). Nonetheless, LMS1 identified a subset of predominantly MSS tumors that had MSI-like gene expression characteristics and poor-prognostic associations. These metastases were not particularly immunogenic, which suggests that there are cancer cell-intrinsic MSI-like features beyond a high tumor mutational burden that have important clinical implications. An additional feature of this subtype was strong oncogenic gene expression signaling, including in the MAPK pathway, and a high relative frequency of *RAS*/*TP53* co-mutations. Such co-mutations have recently been demonstrated to be associated with a poor patient survival after surgical resection of metastatic CRC [41, 42], but the transcriptomic LMS1 group had additional prognostic value independent of the co-mutations.

Pronounced intra-patient inter-metastatic heterogeneity of transcriptomic subtypes was found by analysis of multiple distinct metastatic lesions from a subset of the patients. However, the frequency of heterogeneity varied among the LMS groups, and the least biologically distinct subtypes were also the most heterogeneous. In particular, LMS2 and LMS3 were almost exclusively found intermixed with CRLMs of other subtypes (with the exception of one patient homogenous for LMS3). Accordingly, these subtypes might be particularly sensitive to sampling bias, and this might have contributed to the poor reproducibility in external datasets, each of which included a single-sample/lesion per patient. However, it is likely that these subtypes are too similar or transitory to provide useful stratification of CRLMs. Notably, the distinct LMS1-secretory/MSI-like group was clearly the least heterogeneous subtype in inter-metastatic comparisons, and its prognostic value was consistent in the context of tumor heterogeneity. Although the latter analysis was not conclusive due to the small sample size, it indicates that a single LMS1 metastasis is sufficient to confer a poor patient prognosis after liver resection and standard perioperative treatment, and identifies an important patient population in need of new treatment strategies. Pre-clinical studies have shown that gene/protein expression is the molecular level with the strongest predictive power for drug sensitivities [43], and we have recently shown co-variation among pharmacological and transcriptomic profiles of PDOs from resected CRLMs [25]. Altogether, this supports that LMS1 CRLM poses a new opportunity for rational drug development strategies, although its prognostic association needs to be validated in larger patient series and clinical trial populations.

A limiting factor of this study is the inclusion only of resectable CRLMs, which represent a skewed selection of metastatic CRCs for important molecular factors, such as MSI and *BRAF*^V600E^ mutations. Furthermore, in accordance with current guidelines for the management of patients with resectable or potentially resectable CRLMs [44] most of the samples (from 77% of the patients) had been exposed to neoadjuvant chemotherapy. We observed an impact of neoadjuvant treatment on the gene expression profiles of the samples, consistent with the shift towards a more mesenchymal phenotype enriched with signals of angiogenesis and hypoxia that has previously been shown in treatment-exposed tumors [12]. Most of the tumors that classified as LMS5-mesenchymal in the in-house series were treatment-exposed, but this was not equally prominent in the external dataset, and there was a fair distribution of samples according to treatment status among the epithelial-like subtypes. Furthermore, the main distinction of metastatic CRCs into epithelial-like versus mesenchymal-like tumors has been found both for liver and lung metastases, and shown to be independent of treatment exposure [12]. In primary CRC, such an epithelial-mesenchymal distinction is strongly prognostic, likely reflecting the metastasis promoting effect of TGFβ activation and a contribution from the tumor stroma in the mesenchymal subgroup [1]. Among the CRLMs analyzed here, there was no prognostic difference according to epithelial-like versus mesenchymal characteristics, although LMS5-mesenchymal CRLMs were predominantly diagnosed as synchronous metastases. Notably, the mesenchymal phenotype of LMS5 CRLMs was accompanied by strong immune signals, which are likely to be associated with a favorable patient outcome [45].

## Conclusions

LMS is a metastasis-oriented gene expression-based subtyping framework of CRC that identifies clinically relevant biological traits also in the context of inter-metastatic heterogeneity. Clinical relevance was illustrated by an independent poor-prognostic association of one of the five subtypes, for which a mini-classifier was developed to facilitate prognostic stratification and further clinical testing. In acknowledgment of the weaker reproducibility of some of the subtypes in external CRLM sample series, a consensus framework modeled after the work of the CRC Subtyping Consortium for primary CRCs [3] might be needed to determine the optimal number and frequency of metastasis subtypes. Here, we publish the first large cohort of multi-sample metastatic gene expression profiles, including detailed clinical information and survival data, for future multi-center studies (GSE159216).

## Supplementary Information

Additional file 1: R codes for the data analyses. **Fig. S1.** Overview of study material and analyses. **Fig. S2.** The impact of batch correction on sample type comparisons. **Fig. S3.** Association between selected clinical parameters and gene expression profiles of CRLMs. **Fig. S4.** CMS subtyping of CRLMs using the tailored CMS classifier. **Fig. S5.** GSEA of the epithelial versus mesenchymal subtype from K = 2 factorization. **Fig. S6.** PCA and liver score distribution among LMS groups. **Fig. S7.** Selected single-sample GSVA scores across the LMS groups. **Fig. S8.** GSEA in CRLMs with *RAS/TP53* co-mutations. **Fig. S9.** Kaplan–Meier plots of 5-year CSS according to LMS and translated CMS subtypes. **Fig. S10.** Kaplan–Meier plots of 5-year OS and CSS according to epithelial and mesenchymal subtypes. **Fig. S11.** Kaplan–Meier plots of 5-year OS and CSS according to LMS and *TP53*/*RAS* co-mutations. **Fig. S12.** CRIS classification of the in-house CRLM samples. **Fig. S13.** GSEA of CRLMs in two external datasets according to LMS. **Fig. S14.** De novo transcriptomic subtyping by unsupervised NMF of the two external CRLM datasets. **Fig. S15.** Kaplan–Meier curves of OS and CSS according to LMS1 and tumor heterogeneity. **Fig. S16.** LMS1 mini-classifier is correlated with signatures of LMS1 characteristics. **Fig. S17.** LMS1 mini-classifier captures the poor-prognostic value of LMS1.

Additional file 2: **Table S1.** Association of clinical variables with PC1 and the liver score of CRLMs. **Table S2.** Genes (n = 514) used as input for NMF classification of CRLMs. **Table S3.** GSEA results based on NMF classification at K = 2. **Table S4.** GSEA result for the de novo LMS groups (at NMF K = 5). **Table S5.** Associations of the LMS subtypes (grouped) with mutations and selected clinical variables. **Table S6:** Associations of the LMS groups with clinical variables. **Table S72.** Univariable Cox Proportional hazard analysis. **Table S8.** Multivariable Cox Proportional hazard analysis. **Table S9.** Clinical information of GSE131418 dataset according to LMS subtypes. **Table S10.** Predictive RF model specification.

## Data Availability

Gene expression data from the colorectal liver metastases (*n* = 283), including clinicopathological annotations and patient survival data have been deposited to the GEO under accession number GSE159216 (https://www.ncbi.nlm.nih.gov/geo/query/acc.cgi?acc=GSE159216) [46]. Gene expression profiles of primary CRCs (*n* = 170; GSE96528 available from https://www.ncbi.nlm.nih.gov/geo/query/acc.cgi?acc=GSE96528 and GSE79959 available from https://www.ncbi.nlm.nih.gov/geo/query/acc.cgi?acc=GSE79959) and CRC cell lines (*n* = 34; GSE97023 available from https://www.ncbi.nlm.nih.gov/geo/query/acc.cgi?acc=GSE97023) have previously been published [6, 24, 26]. The external gene expression datasets of liver metastases were downloaded from GSE131418 (*n* = 141; MCC dataset; available at https://www.ncbi.nlm.nih.gov/geo/query/acc.cgi?acc=GSE131418 [12]) and GSE73255 (*n* = 167 available from https://www.ncbi.nlm.nih.gov/geo/query/acc.cgi?acc=GSE73255 [4]). All data analyses were performed in R and all data and codes essential to evaluate the conclusions in the paper are presented in the paper and/or the Supplementary Materials.

## Abbreviations

CI: Confidence interval
CMS: Consensus molecular subtype
CRC: Colorectal cancer
CRIS: CRC intrinsic subtypes
CRLM: CRC liver metastases
CSS: Cancer-specific survival
GEO: Gene expression omnibus
GSEA: Gene set enrichment analysis
GSVA: Gene set variation analysis
HGNC: HUGO Gene Nomenclature Committee
HR: Hazard ratio
HTA 2.0: Human Transcriptome Array 2.0
LMS: Liver metastasis subtype
MSI: Microsatellite instability
NMF: Nonnegative matrix factorization
OR: Odds ratio
OS: Overall survival
PC1: First principal component
PCA: Principal component analysis
PDO: Patient-derived organoids
*RAS*: *KRAS*/*NRAS*
RIN: RNA integrity number
RMA: Robust multi-array average
SD: Standard deviation
