# Supplementary material for: De novo transcriptomic subtyping of colorectal cancer liver metastases in the context of tumor heterogeneity
Source: Genome Med. 2021 Sep 1;13:143. doi: 10.1186/s13073-021-00956-1 (PMC8411513; doi:10.1186/s13073-021-00956-1)
Supplement: Supplementary file 1 — R codes for the data analyses. Fig. S1. Overview of study material and analyses. Fig. S2. The impact of batch correction on sample type comparisons. Fig. S3. Association between selected clinical parameters and gene expression profiles of CRLMs. Fig. S4. CMS subtyping of CRLMs using the tailored CMS classifier. Fig. S5. GSEA of the epithelial versus mesenchymal subtype from K = 2 factorization. Fig. S6. PCA and liver score distribution among LMS groups. Fig. S7. Selected single-sample GSVA scores across the LMS groups. Fig. S8. GSEA in CRLMs with RAS/TP53 co-mutations. Fig. S9. Kaplan–Meier plots of 5-year CSS according to LMS and translated CMS subtypes. Fig. S10. Kaplan–Meier plots of 5-year OS and CSS according to epithelial and mesenchymal subtypes. Fig. S11. Kaplan–Meier plots of 5-year OS and CSS according to LMS and TP53/RAS co-mutations. Fig. S12. CRIS classification of the in-house CRLM samples. Fig. S13. GSEA of CRLMs in two external datasets according to LMS. Fig. S14. De novo transcriptomic subtyping by unsupervised NMF of the two external CRLM datasets. Fig. S15. Kaplan–Meier curves of OS and CSS according to LMS1 and tumor heterogeneity. Fig. S16. LMS1 mini-classifier is correlated with signatures of LMS1 characteristics. Fig. S17. LMS1 mini-classifier captures the poor-prognostic value of LMS1. [file 13073_2021_956_MOESM1_ESM.pdf]

Additional file 1: R code and Supplementary Figures for

De novo transcriptomic subtyping of colorectal cancer liver metastases in the context of tumor heterogeneity

Seyed H. Moosavi, Peter W. Eide, Ina A. Eilertsen, Tuva H. Brunsell, Kaja C. G. Berg, Bård I. Røsok, Kristoffer W. Brudvik, Bjørn A. Bjørnbeth, Marianne G. Guren, Arild Nesbakken, Ragnhild A. Lothe, Anita Sveen

R codes for the data analyses

```
library(knitr)
library(readr)
library(affy)
library(org.Hs.eg.db)
library(matrixStats)
library(limma)
library(GSVA)
library(CMScaller)
library(scales)
library(factoextra)
library(NMF)
library(doMC)
library(dplyr)
library(caret)
library(randomForest)

load("./RData/knit_LMS_subtypes_v2.RData")
```

This is an R Markdown document (http://rmarkdown.rstudio.com).

```
#Title: de novo transcriptomic subtype discovery of colorectal cancer liver m
etastases (CRLMs)

#STEP1: Cancer-specific genes identified by differential gene expression analysis (LFC=0, P-val
= 0.05) between CRLMs and adjacent normal liver samples
# MatA = Gene Expression matrix of 169 unique CRLMs and 19 adjacent normal liver samples normal
ized together by
#justRMA(filenames = "NameOfCEL", celfile.path = "pathToCELFiles", normalize = TRUE, cdfname =
"hta20hsentrezgcdf")
# gene.list = List of genes of interest from BioMart database (genes annotated as protein-codin
g, lincRNA, miRNA)
#info = A data.frame with all samples in MatA in rows and "Status" column indicating whether th
e sample is "CRLM" or "Normal liver"

head(gene.list)

## [1] "A1BG"    "A1CF"    "A2M"     "A2ML1"   "A3GALT2" "A4GALT"

length(gene.list)

[1] 23464

table(info$Status)

##
##        CRLM NormalLiver
##         169          19


MetNor

## ExpressionSet (storageMode: lockedEnvironment)
## assayData: 32496 features, 188 samples
##   element names: exprs, se.exprs
## protocolData
##   sampleNames: patient_CRLM1 patient_CRLM2 ... patient_CRLM188 (188
##     total)
```

```
##   varLabels: ScanDate
##   varMetadata: labelDescription
## phenoData
##   sampleNames: patient_CRLM1 patient_CRLM2 ... patient_CRLM188 (188
##     total)
##   varLabels: sample
##   varMetadata: labelDescription
## featureData: none
## experimentData: use 'experimentData(object)'
## Annotation: hta20hsentrezgcdf

MatA <- exprs(MetNor)
rownames(MatA) <- gsub("_at", "", rownames(MatA)) #Remove control probes
row.names(MatA) <- unname(annotate::getSYMBOL(row.names(MatA),"org.Hs.eg.db")) # Convert row na
mes from ENTREZ ID to GENE SYMBOL
MatA <- MatA[row.names(MatA) %in% gene.list,] #Select genes of interest

#RUN DIFFERENTIAL GENE EXPRESSION ANALYSIS
DEGs <- CMScaller::subDEG(MatA, factor(info$Status,c("CRLM","NormalLiver")), padj = 0.05, sortB
y = "p", doPlot = TRUE)

## contrast is CRLM vs NormalLiver

DEGs <- DEGs[DEGs$adj.P.Val < 0.05 & DEGs$logFC > 0,]
head(DEGs)

##             logFC   AveExpr        t      P.Value    adj.P.Val        B
## LGALS3   3.303604 10.003474 30.71956 1.747266e-75 1.465822e-72 161.7239
## ARPC2    1.534109  9.939466 28.30421 5.992579e-70 2.055724e-67 149.0286
## SEMA3C   3.763182  8.029953 28.12587 1.577725e-69 5.214142e-67 148.0639
## SLC25A24 2.268942  7.694760 26.93671 1.105933e-66 2.593829e-64 141.5327
## FAR1     2.750861  8.355547 26.50634 1.235867e-65 2.779045e-63 139.1263
## SLC25A36 2.455936  7.522680 26.34749 3.029658e-65 6.542861e-63 138.2322

DEGs <- as.character(rownames(DEGs))
length(DEGs)

## [1] 6247

head(DEGs)

## [1] "LGALS3"   "ARPC2"    "SEMA3C"   "SLC25A24" "FAR1"     "SLC25A36"



#STEP2: Filtering of cancer-specific genes to include only genes with high sample-vise variatio
n among CRLMs (SD > 0.8)
#MatB = Gene Expression matrix of 169 unique CRLMs normalized by
#justRMA(filenames = "NamedOfCEL", celfile.path = "pathToCELFiles", normalize = TRUE, cdfname =
 "hta20hsentrezgcdf" )

Meteset

## ExpressionSet (storageMode: lockedEnvironment)
## assayData: 32496 features, 169 samples
##   element names: exprs, se.exprs
## protocolData
##   sampleNames: patient_CRLM1 patient_CRLM2 ... patient_CRLM169 (169
##     total)
##   varLabels: ScanDate
##   varMetadata: labelDescription
## phenoData
```

```
##   sampleNames: patient_CRLM1 patient_CRLM2 ... patient_CRLM169 (169
##     total)
##   varLabels: sample
##   varMetadata: labelDescription
## featureData: none
## experimentData: use 'experimentData(object)'
## Annotation: hta20hsentrezgcdf

MatB <- exprs(Meteset)
dim(MatB)

## [1] 32496   169

rownames(MatB) <- gsub("_at", "", rownames(MatB))
row.names(MatB) <- unname(annotate::getSYMBOL(row.names(MatB),"org.Hs.eg.db")) # Covert row nam
es from ENTREZ ID to GENE SYMBOL
MatB <- MatB[row.names(MatB) %in% DEGs,] # Select the cancer specific genes

MatB.genes <- rownames(MatB[which(rowSds(MatB) > 0.8),])  # Select genes with high SD

length(MatB.genes)

## [1] 514

MatB.NMF <- exp(MatB[row.names(MatB) %in% MatB.genes,]) # Select genes with SD > 0.8 as a matri
x with exponential transformation
dim(MatB.NMF)

## [1] 514 169

#STEP3: Run NMF
#Perform NMF with factorization level 2 to 6
preNMF <- nmf(MatB.NMF,"brunet",seed="random", rank = 2:6, .options = "vP3", nrun=100)
class(preNMF)

## [1] "NMF.rank"

plot(preNMF,main="NMF 2:6 statistics")
```

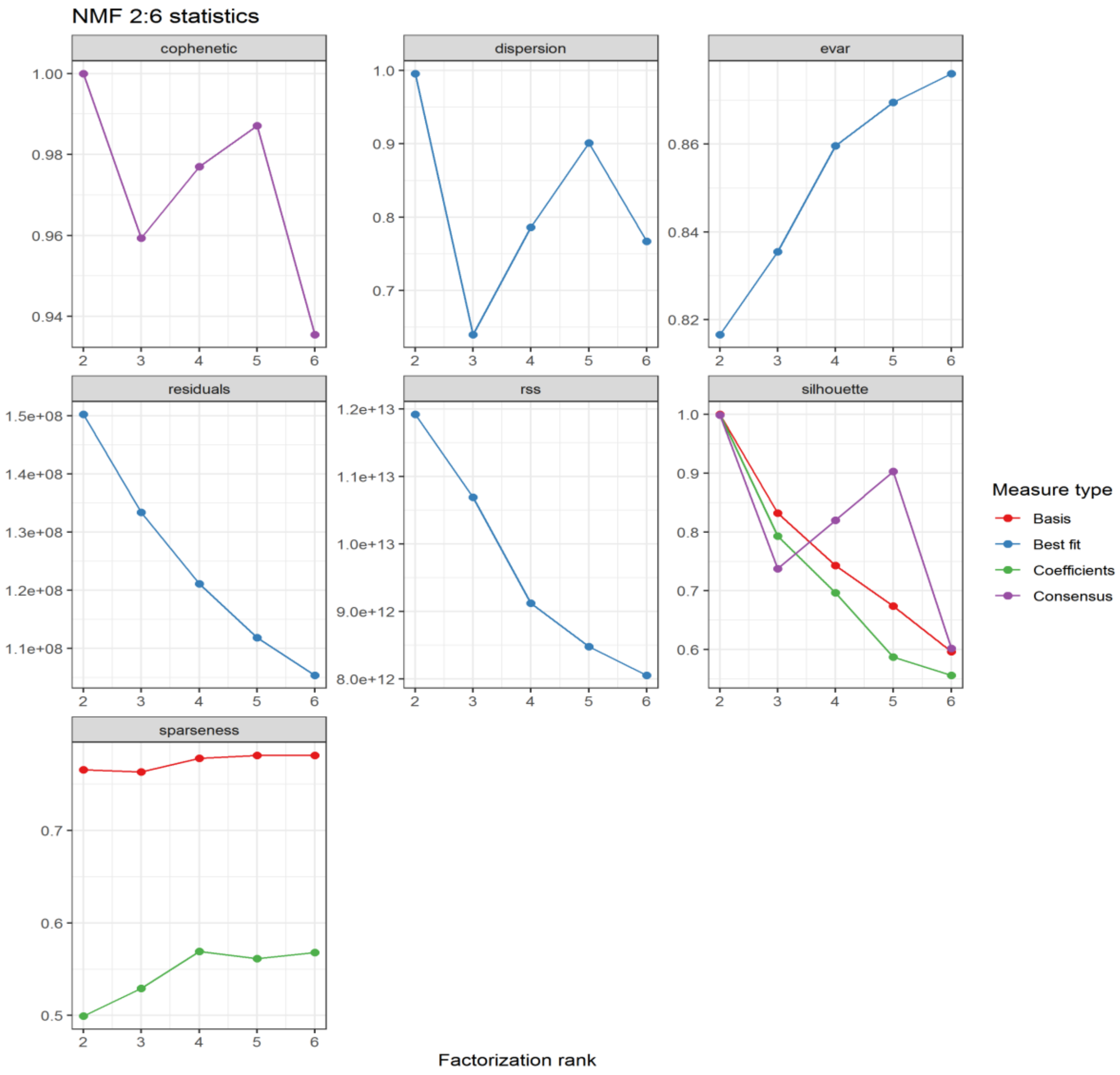


```
consensusmap(preNMF)
```

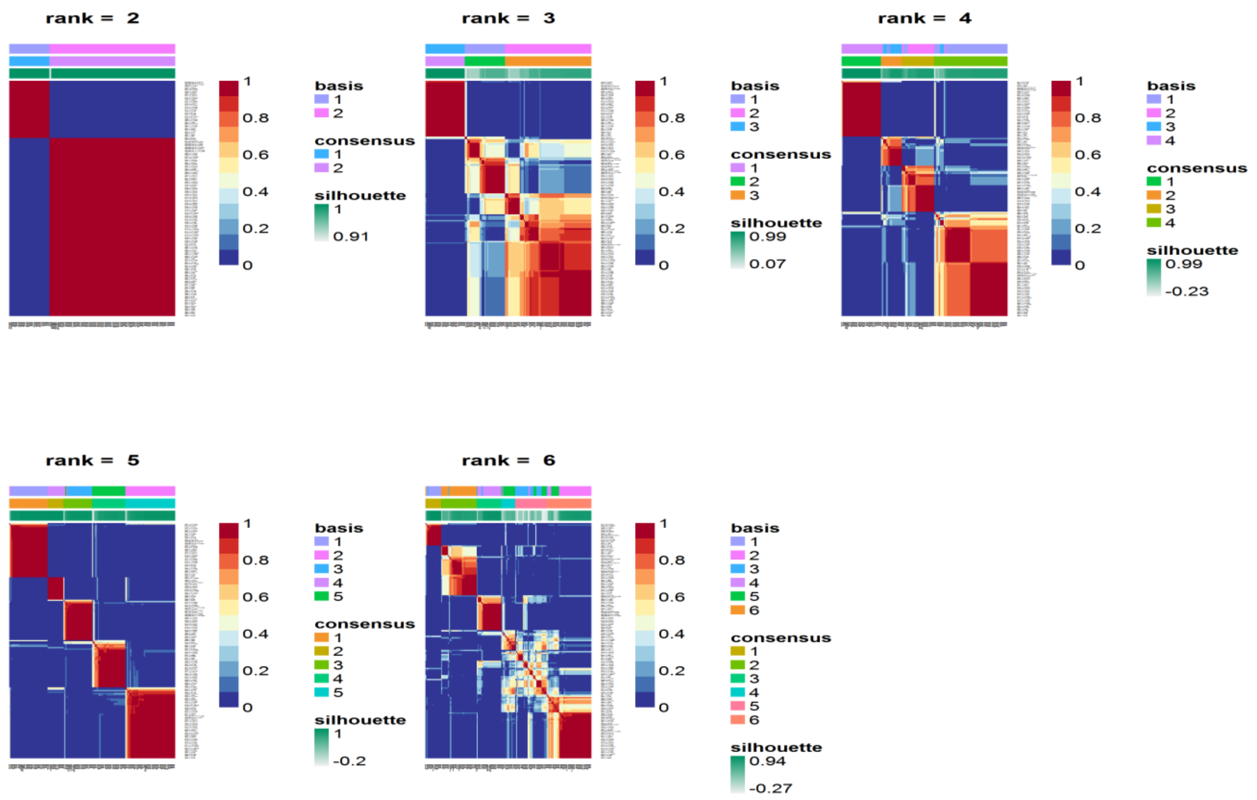


```
#NMF classification at k=2

usedrngk2 #Used seed for classification at k=2

## [1]         407    82000378 -1377755341   -888237352   -352809223   1464214118
## [7] -1790002769

NMF_2 <- nmf(MatB.NMF,"brunet",seed="random", rank = 2, .options = "vP3", nrun=100, rng=usedrng
k2)

## NMF algorithm: 'brunet'

## Multiple runs: 100

## Mode: parallel (3/4 core(s))

## Runs: |                                                                 Runs: |
      |   0%Runs: |  Runs: |==================================================| 100%
## System time:
##    user  system elapsed
##    1.43    0.08   26.42

#NMF classification at k=5

usedrngk5 #Used seed for classification at k=5

## [1]         407 -1167312791  -382305174  1385648097  1560322171  1326776643
## [7] -1344225789
```

```
NMF_5 <- nmf(MatB.NMF,"brunet",seed="random", rank = 5, .options = "vP3", nrun=100, rng=usedrng
k5) # Perform NMF at k=5

## NMF algorithm: 'brunet'

## Multiple runs: 100

## Mode: parallel (3/4 core(s))

## Runs: |                                                                          Runs: |
                                  |    0%Runs: |   Runs: |==============================================
=====| 100%
## System time:
##    user  system elapsed
##    1.32    0.16  126.97

Mycol3 <- c("firebrick3","deepskyblue1","lightcoral","forestgreen","darkorange2")

plot(silhouette(NMF_5),col=Mycol3, main="silhouette width")
```

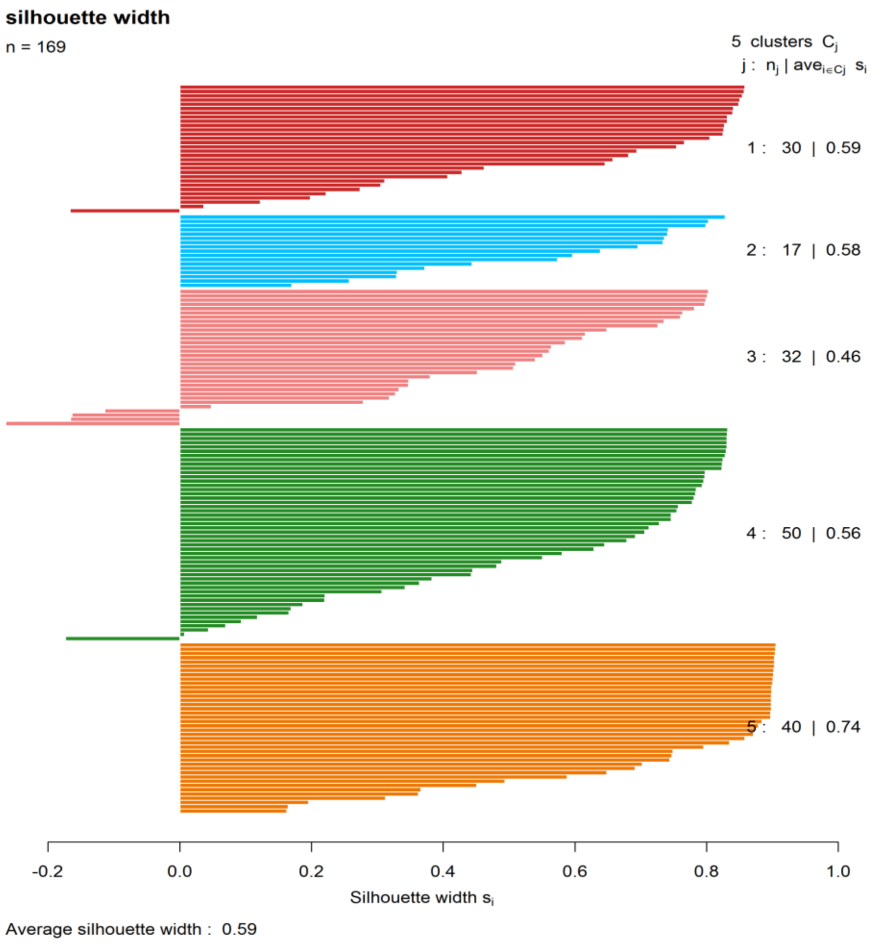


```
#Result of the NMF classification
table(paste0("LMS",predict(NMF_5, "columns")))

##
## LMS1 LMS2 LMS3 LMS4 LMS5
##   30   17   32   50   40
```

```r
#Comparison of the NMF results at K=2 and K=5
table(NMF_K5=predict(NMF_5,"columns"), NMF_K2=predict(NMF_2,"columns"))

##        NMF_K2
## NMF_K5  1  2
##      1  0 30
##      2  1 16
##      3  1 31
##      4  0 50
##      5 39  1

#STEP4: Perform Gene Set Enrichment Analysis (GSEA)
#Geneset = list of gene sets to be used for GSEA

MatB <- exprs(Meteset)
rownames(MatB) <- gsub("_at", "", rownames(MatB))
row.names(MatB) <- unname(annotate::getSYMBOL(row.names(MatB),"org.Hs.eg.db"))

names(Genesets) <- tools::toTitleCase(names(Genesets))
heatcol <- rev(grDevices::colorRampPalette(RColorBrewer::brewer.pal(6, "RdBu"))(12))
col <- grDevices::colorRampPalette(RColorBrewer::brewer.pal(9,"RdYlGn"))(100)
colh <- grDevices::colorRampPalette(RColorBrewer::brewer.pal(6,"Spectral"))(100)


par(mar=c(5, 15, 4, 2),font=1, cex=0.7, font.sub=2)
GSEAres <- CMScaller::subCamera(MatB, paste0("LMS",predict(NMF_5, "columns")) , geneList = Gene
sets, heatCol=heatcol, classCol = Mycol3, topN = 40, rowCluster = T, main="Top 30 gene sets")
```

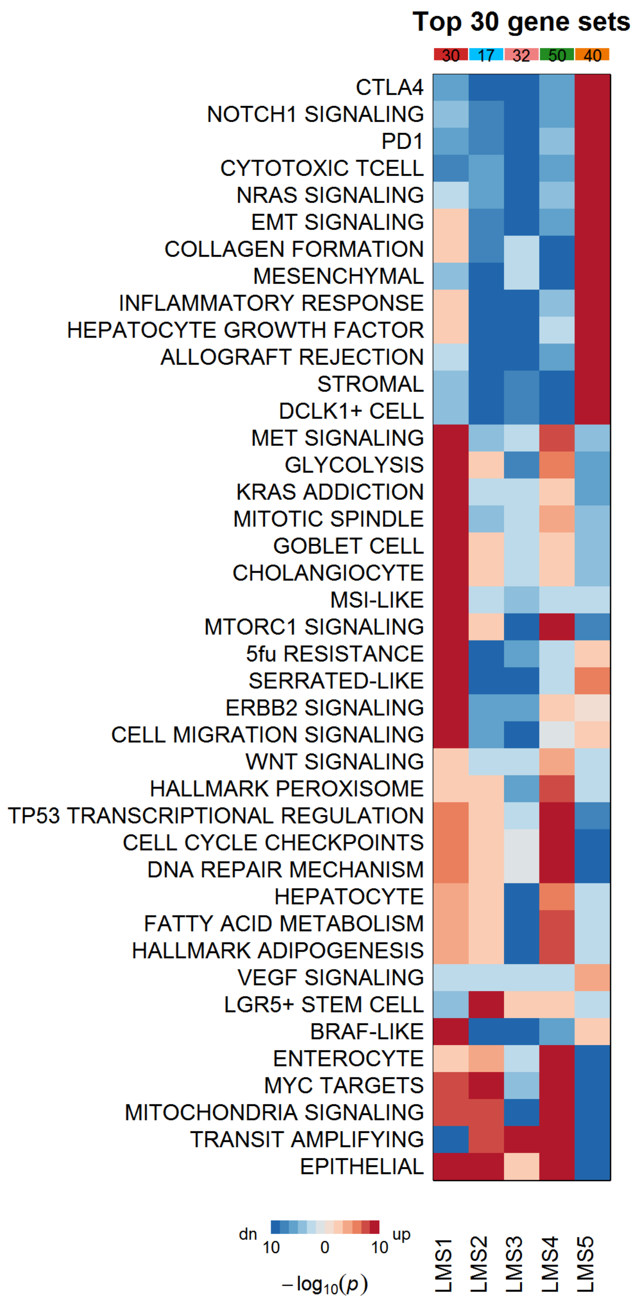
Top 30 gene sets
30
17
32
50
40
CTLA4
NOTCH1 SIGNALING
PD1
CYTOTOXIC TCELL
NRAS SIGNALING
EMT SIGNALING
COLLAGEN FORMATION
MESENCHYMAL
INFLAMMATORY RESPONSE
HEPATOCYTE GROWTH FACTOR
ALLOGRAFT REJECTION
STROMAL
DCLK1+ CELL
MET SIGNALING
GLYCOLYSIS
KRAS ADDICTION
MITOTIC SPINDLE
GOBLET CELL
CHOLANGIOCYTE
MSI-LIKE
MTORC1 SIGNALING
5fu RESISTANCE
SERRATED-LIKE
ERBB2 SIGNALING
CELL MIGRATION SIGNALING
WNT SIGNALING
HALLMARK PEROXISOME
TP53 TRANSCRIPTIONAL REGULATION
CELL CYCLE CHECKPOINTS
DNA REPAIR MECHANISM
HEPATOCYTE
FATTY ACID METABOLISM
HALLMARK ADIPOGENESIS
VEGF SIGNALING
LGR5+ STEM CELL
BRAF-LIKE
ENTEROCYTE
MYC TARGETS
MITOCHONDRIA SIGNALING
TRANSIT AMPLIFYING
EPITHELIAL
dn
up
10
0
10
$-\log_{10}(p)$
LMS1
LMS2
LMS3
LMS4
LMS5

```
#Title: LMS random forest class prediction model

MatB <- exprs(Meteset)
dim(MatB)

[1] 32496   169

rownames(MatB) <- gsub("_at", "", rownames(MatB))
row.names(MatB) <- unname(annotate::getSYMBOL(row.names(MatB),"org.Hs.eg.db")) # Covert row nam
es from ENTREZ ID to GENE SYMBOL
MatB <- MatB[row.names(MatB) %in% DEGs,] # Select the results of differential expression analys
is
MatB.genes <- rownames(MatB[which(rowSds(MatB) > 0.8),])  # Select genes with SD > 0.8 as a lis
t
length(MatB.genes)

[1] 514

nos <- unname(which(silhouette(NMF_5)[,3] < .0 )) # samples with negative silhouette width for
NMF K=5
MatB <- MatB[row.names(MatB) %in% MatB.genes,-nos] # Select genes with SD > 0.8 as a matrix
dim(MatB)

[1] 514 163

#Train Random forest model
library(caret)
set.seed(69)
trainctrl <- trainControl(method = "repeatedcv",number = 7, repeats = 1, returnResamp = "all",
savePredictions = "all",
                          classProbs = TRUE,
                          summaryFunction = multiClassSummary,
                          returnData = TRUE, search = "random")
control <- rfeControl(functions=rfFuncs, method = "repeatedcv", number= 7,
                      repeats = 1 , verbose=F, allowParallel=TRUE, saveDetails=T, p = 1,rerank=
TRUE)



rfe.feature <- rfe(t(MatB), y = factor(paste0("LMS",predict(NMF_5, "columns")))[-nos],
                   sizes=seq(100,700,10), method = "parRF",trControl = trainctrl ,
                   rfeControl=control, metric="Accuracy")


print(rfe.feature)

Recursive feature selection
Outer resampling method: Cross-Validated (7 fold, repeated 1 times)
Resampling performance over subset size:
 Variables Accuracy  Kappa AccuracySD KappaSD Selected
       100   0.7365 0.6514    0.12528 0.16656
       110   0.7668 0.6909    0.10603 0.14182
       120   0.7544 0.6739    0.09335 0.12590
       130   0.7425 0.6569    0.10227 0.13725
       140   0.7787 0.7071    0.10553 0.14087
       150   0.7790 0.7073    0.06883 0.09150
       160   0.7790 0.7073    0.09889 0.13307
       170   0.7544 0.6748    0.08634 0.11582
       180   0.7852 0.7153    0.07918 0.10525        *
       190   0.7666 0.6907    0.10370 0.13835
       200   0.7725 0.6973    0.08701 0.11692
```

```
       210   0.7728 0.6987    0.07851 0.10371
       220   0.7479 0.6646    0.09957 0.13289
       230   0.7849 0.7139    0.08014 0.10675
       240   0.7787 0.7066    0.08138 0.10787
       250   0.7723 0.6979    0.10092 0.13372
       260   0.7663 0.6891    0.08771 0.11654
       270   0.7479 0.6640    0.08958 0.11962
       280   0.7666 0.6903    0.07130 0.09539
       290   0.7601 0.6808    0.08791 0.11761
       300   0.7601 0.6810    0.08791 0.11773
       310   0.7601 0.6814    0.09480 0.12663
       320   0.7604 0.6820    0.09763 0.13022
       330   0.7539 0.6730    0.06724 0.08991
       340   0.7787 0.7064    0.10250 0.13695
       350   0.7785 0.7045    0.09335 0.12575
       360   0.7539 0.6722    0.09111 0.12141
       370   0.7541 0.6724    0.08682 0.11660
       380   0.7539 0.6725    0.10095 0.13473
       390   0.7668 0.6903    0.07464 0.09942
       400   0.7477 0.6636    0.09031 0.12140
       410   0.7479 0.6656    0.08256 0.11109
       420   0.7723 0.6972    0.08387 0.11338
       430   0.7663 0.6889    0.09124 0.12262
       440   0.7544 0.6721    0.08198 0.11035
       450   0.7541 0.6739    0.08342 0.11214
       460   0.7666 0.6887    0.07932 0.10606
       470   0.7663 0.6893    0.07618 0.10081
       480   0.7541 0.6722    0.07923 0.10569
       490   0.7541 0.6726    0.08682 0.11607
       500   0.7539 0.6724    0.09111 0.12193
       510   0.7598 0.6813    0.06829 0.09075
       514   0.7536 0.6713    0.09494 0.12789
The top 5 variables (out of 180):
   ANXA1, MYO5A, CLIP4, DOCK2, OLFM4

head(print(rfe.feature$optVariables),20)

[1] "ANXA1"   "MYO5A"   "CLIP4"   "DOCK2"   "OLFM4"   "SATB2"   "GCNT3"
 [8] "PIK3CG"  "PLXNC1"  "NCKAP1L" "KCTD12"  "MS4A4A"  "DOCK8"   "CD84"
[15] "TM6SF1"  "CCDC88A" "DOCK10"  "GRM8"    "IRAK3"   "LY6G6D"

table(predict(rfe.feature, t(MatB))[,1],factor(paste0("LMS",predict(NMF_5, "columns")))[-nos])


       LMS1 LMS2 LMS3 LMS4 LMS5
  NMF1   29    0    0    0    0
  NMF2    0   17    0    0    0
  NMF3    0    0   28    0    0
  NMF4    0    0    0   49    0
  NMF5    0    0    0    0   40

head(varImp(rfe.feature, value = "randomForest"),20)

         Overall
ANXA1   4.028922
MYO5A   3.820845
CLIP4   3.531559
OLFM4   3.474523
DOCK2   3.377483
SATB2   3.111687
GCNT3   2.647251
PIK3CG  2.617658
```

```
MS4A4A   2.547657
KCTD12   2.400621
NCKAP1L  2.257365
CD84     2.108349
DOCK8    2.086296
PLXNC1   2.007672
DOCK10   1.970468
TM6SF1   1.966743
CCDC88A  1.895612
RUBCNL   1.881159
SLC1A3   1.842757
CLEC7A   1.839537

#Title: Development of LMS1 "mini-classifier"
MatB <- exprs(Meteset)
dim(MatB)

[1] 32496   169

rownames(MatB) <- gsub("_at", "", rownames(MatB))
row.names(MatB) <- unname(annotate::getSYMBOL(row.names(MatB),"org.Hs.eg.db")) # Convert row na
mes from ENTREZ ID to GENE SYMBOL
MatB <- MatB[row.names(MatB) %in% gene.list,]

#Preparing a data frame with CRLMs classified as either LMS1 or LMS2-5
MatBcolnames <- as.character(sub("\\(2)_|_$","",gsub("_Rep|.{14}$|\\(HTA.*","",colnames(MatB)))
)
info2 <- na.omit(info[match(MatBcolnames,info$sample),] )
all.equal(info2$sample,MatBcolnames)

[1] TRUE

info2$class <- paste0("LMS",predict(NMF_5, "columns"))
info2$class <- ifelse(grepl("LMS1",info2$class),"Poor","Good") #changing labels to LMS1=poor an
d LMS2-5=Good based on the survival
table(info2$class)
Good Poor
 139   30

#DEG between LMS1 and LMS2-5
mini_design <- model.matrix(~0+factor(info2$class))
colnames(mini_design) <- c("Good","Poor")
row.names(mini_design) <- row.names(info2)
mini_fitMet <- lmFit(MatB, mini_design)

mini_cont <- makeContrasts(LMS1vsR = Poor-Good,
                      levels=mini_design)
mini_cont

      Contrasts
Levels LMS1vsR
  Good      -1
  Poor       1

#perform DEG using Limma
mini_fitMet2 <- contrasts.fit(mini_fitMet, mini_cont)
mini_fitMet2 <- eBayes(mini_fitMet2, robust=TRUE)
mini_DEGs <- rownames(topTable(mini_fitMet2,coef = 1 , number = nrow(mini_fitMet2), adjust.meth
od="BH",
                              p.value=0.05, sort.by="p", lfc=1.6, resort.by= "B"))
length(mini_DEGs)
```

```
[1] 9

print(mini_DEGs) #genes used for training of the mini-classifier

[1] "GCNT3"    "CTSE"     "REG4"     "TCN1"     "LCN2"     "DSG3"     "UCA1"
[8] "SERPINB5" "MUC17"

LMS1exp <- MatB[rownames(MatB) %in% mini_DEGs,] #A gene expression matrix with the 9 genes from
 DEG
dim(LMS1exp)

[1]   9 169

mini_trainctrl <- trainControl(method = "LOOCV",
                          number = 7, repeats = 1,
                          classProbs = TRUE,
                          summaryFunction = twoClassSummary,
                          savePredictions="final",
                          search = "random")

# PARAMTER TUNING
set.seed(1111111)
mini_RF  <- train(x = t(LMS1exp), y = factor(info2$class),
                  method = "parRF",
                  trControl = mini_trainctrl,
                  metric = "ROC",
                  tuneLength = 10)

#TUNED PARAMETRS
getModelInfo(model = "parRF", regex = FALSE)[[1]]$parameter #Replace with the tuned parameters
mini_tGrid <-  expand.grid(mtry = mini_RF$bestTune[1,1])
set.seed(1111111)
mini_RF_mod <- train(t(LMS1exp), y = factor(info2$class),
                  trControl = mini_trainctrl,
                  tuneGrid = mini_tGrid,
                  method = "parRF",
                  metric = "ROC")


mini_RF_mod$call

train.default(x = t(LMS1exp), y = factor(info$survival), method = "parRF",
    metric = "ROC", trControl = mini_trainctrl, tuneGrid = mini_tGrid)

print(mini_RF_mod)

Parallel Random Forest
169 samples
  9 predictor
  2 classes: 'Good', 'Poor'
No pre-processing
Resampling: Leave-One-Out Cross-Validation
Summary of sample sizes: 168, 168, 168, 168, 168, 168, ...
Resampling results:

  ROC        Sens       Spec
  0.9416067  0.9856115  0.6666667

Tuning parameter 'mtry' was held constant at a value of 2

table(predict(mini_RF_mod, t(LMS1exp)),info2$class)
```

```
      Good Poor
 Good  139    0
 Poor    0   30
```

```r
sessionInfo()
```

```
R version 3.5.1 (2018-07-02)
Platform: x86_64-w64-mingw32/x64 (64-bit)
Running under: Windows 7 x64 (build 7600)

Matrix products: default

locale:
[1] LC_COLLATE=English_United Kingdom.1252
[2] LC_CTYPE=English_United Kingdom.1252
[3] LC_MONETARY=English_United Kingdom.1252
[4] LC_NUMERIC=C
[5] LC_TIME=English_United Kingdom.1252

attached base packages:
[1] stats4    parallel  stats     graphics  grDevices utils     datasets
[8] methods   base

other attached packages:
 [1] doParallel_1.0.15    RColorBrewer_1.1-2   randomForest_4.6-14
 [4] caret_6.0-85         lattice_0.20-41      dplyr_0.8.4
 [7] doMC_1.3.5           iterators_1.0.12     foreach_1.4.8
[10] NMF_0.22.0           cluster_2.1.0        rngtools_1.5
[13] pkgmaker_0.31.1      registry_0.5-1       factoextra_1.0.6
[16] ggplot2_3.3.0        scales_1.1.0         CMScaller_1.9.2
[19] GSVA_1.30.0          limma_3.38.3         matrixStats_0.55.0
[22] org.Hs.eg.db_3.7.0   AnnotationDbi_1.44.0 IRanges_2.16.0
[25] S4Vectors_0.20.1     affy_1.62.0          Biobase_2.42.0
[28] BiocGenerics_0.28.0  readr_1.3.1          knitr_1.28

loaded via a namespace (and not attached):
 [1] colorspace_1.4-1      class_7.3-15          farver_2.0.3
 [4] affyio_1.52.0         ggrepel_0.8.1         bit64_0.9-7
 [7] prodlim_2019.11.13    lubridate_1.7.4       codetools_0.2-16
[10] splines_3.5.1         geneplotter_1.60.0    shinythemes_1.1.2
[13] pROC_1.16.1           gridBase_0.4-7        annotate_1.60.1
[16] graph_1.60.0          shiny_1.4.0           BiocManager_1.30.10
[19] compiler_3.5.1        assertthat_0.2.1      Matrix_1.2-18
[22] fastmap_1.0.1         later_1.0.0           htmltools_0.4.0
[25] tools_3.5.1           gtable_0.3.0          glue_1.3.1
[28] reshape2_1.4.3        Rcpp_1.0.3            vctrs_0.2.3
[31] preprocessCore_1.44.0 nlme_3.1-137          timeDate_3043.102
[34] xfun_0.12             gower_0.2.1           stringr_1.4.0
[37] mime_0.9              lifecycle_0.1.0       statmod_1.4.34
[40] XML_3.99-0.3          zlibbioc_1.28.0       MASS_7.3-51.5
[43] ipred_0.9-9           hms_0.5.3             promises_1.1.0
[46] yaml_2.2.1            memoise_1.1.0         rpart_4.1-15
[49] stringi_1.4.6         RSQLite_2.2.0         e1071_1.7-3
[52] bibtex_0.4.2.2        lava_1.6.6            rlang_0.4.5
[55] pkgconfig_2.0.3       bitops_1.0-6          evaluate_0.14
[58] purrr_0.3.3           recipes_0.1.9         labeling_0.3
[61] import_1.1.0          bit_1.1-15.2          tidyselect_1.0.0
[64] GSEABase_1.44.0       plyr_1.8.5            magrittr_1.5
[67] R6_2.4.1              generics_0.0.2        DBI_1.1.0
[70] pillar_1.4.3          withr_2.1.2           survival_3.1-8
```

```
[73] RCurl_1.98-1.1       nnet_7.3-13          tibble_2.1.3
[76] crayon_1.3.4         rmarkdown_2.3        grid_3.5.1
[79] data.table_1.12.8    blob_1.2.1           ModelMetrics_1.2.2.1
[82] digest_0.6.25        xtable_1.8-4         httpuv_1.5.2
[85] munsell_0.5.0
```

Supplementary Figures

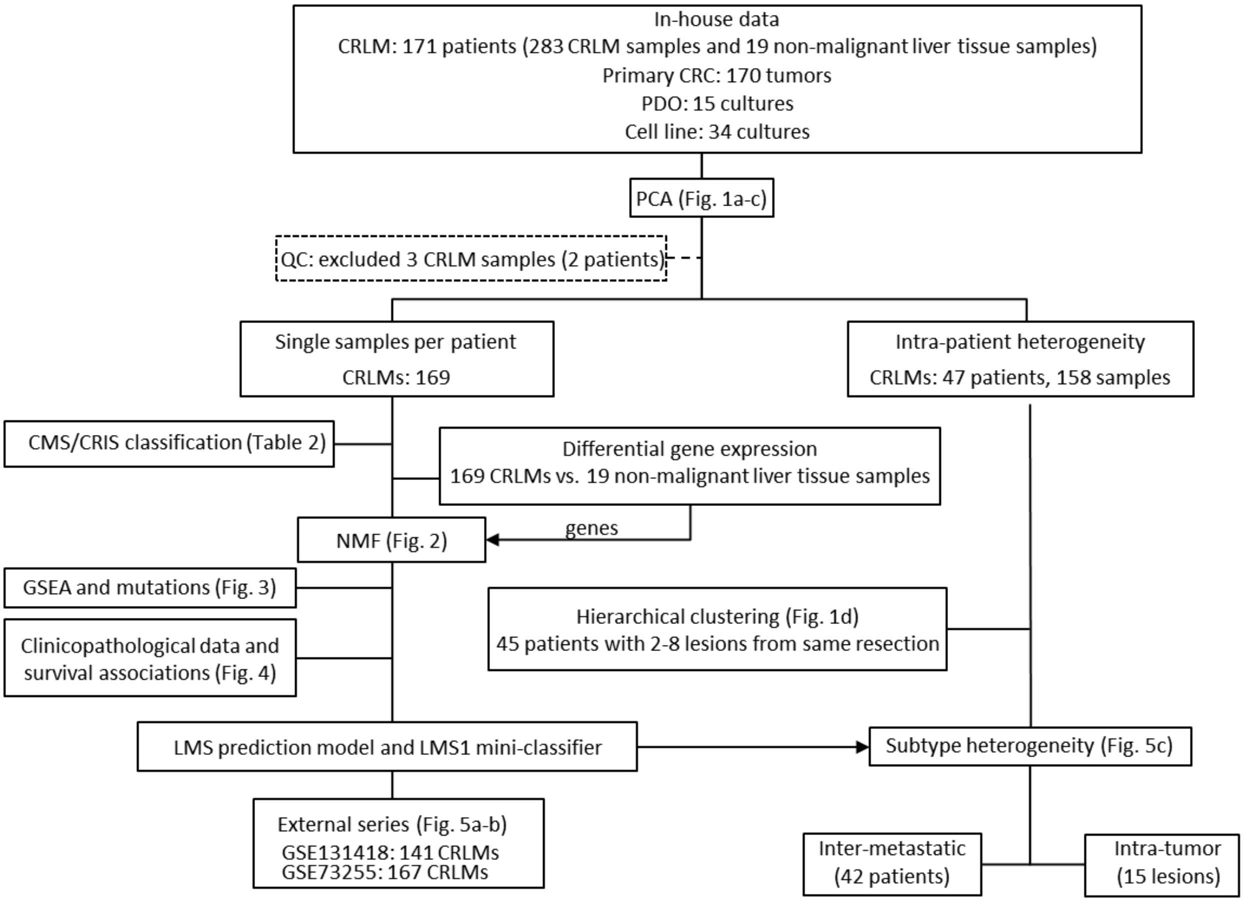

Fig. S1. Overview of study material and analyses.

The diagram indicates sample inclusion in the main analyses presented in the study. The study workflow is presented from top to bottom.

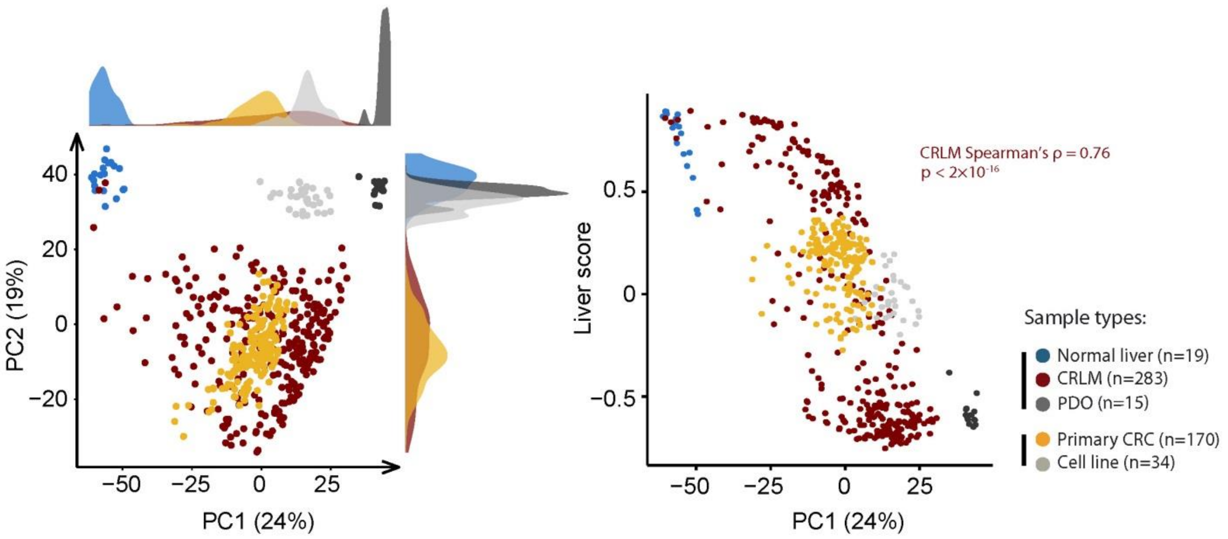

Batch correction	Marker	Group1	Group2	P-value	P-value.adj (BH)	P-value.signif
**No**	***ALB***	CRLM	Primary	7.91E-71	1.60E-69	****
**Yes**	***ALB***	CRLM	Primary	0.24	0.3	Insignificant

Fig. S2 The impact of batch correction on sample type comparisons.

In-house gene expression data were generated in two different batches (batch 1: primary CRCs and cell lines; batch 2: CRLMs, normal liver samples, and PDOs). The plots from PCA shown here are for batch corrected data, and correspond to the plots generated without batch correction in main Fig. 1**a-b**. PCA was performed on the 1000 genes with highest variation across the batch corrected dataset, and the liver score was calculated by GSVA of a set of genes highly expressed in the liver.

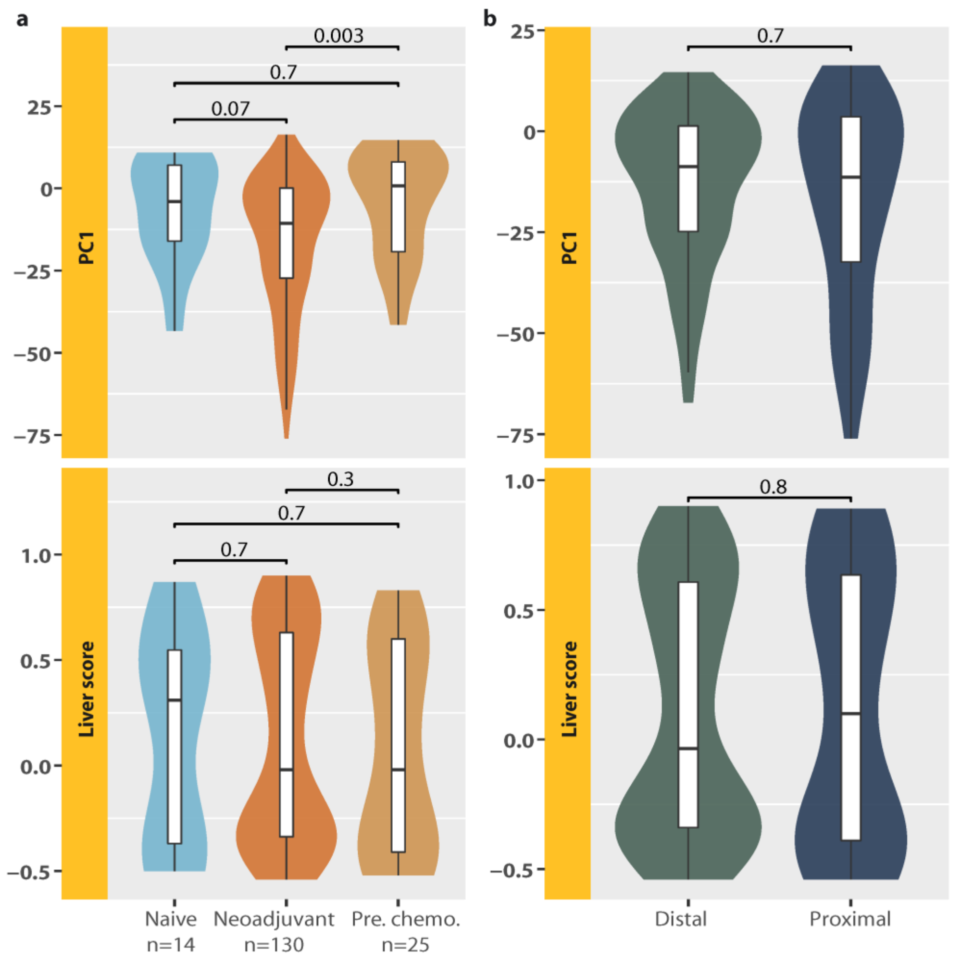

Fig. S3. Association between selected clinical parameters and gene expression profiles of CRLMs.

a Impact of systemic treatment prior to tumor sampling on the gene expression profiles, analyzed as PC1 values (from PCA of the 1000 genes with highest SD across patients) and the liver scores. Neoadjuvant chemotherapy was associated with lower PC1 values. **b** Primary tumor sidedness was not associated with any of the gene expression measures. Wilcoxon test p-values are denoted.

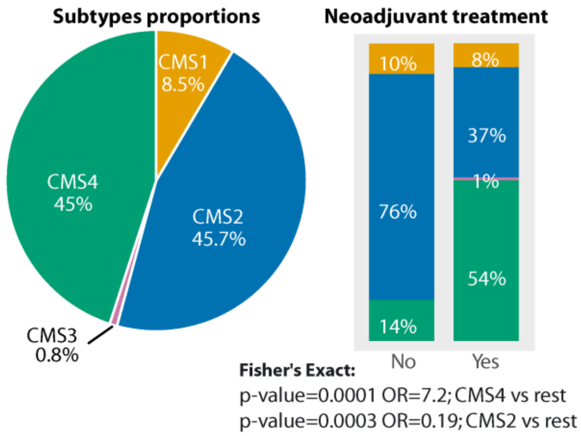

Fig. S4. CMS subtyping of CRLMs using the tailored CMS classifier.

CRLMs were classified according to CMS using our classifier adapted to the liver setting (CMScaller v2.0). Among single CRLMs (randomly selected) from each of the 169 patients, 129 were confidently classified. The pie chart shows the proportion of samples in each CMS group, and the bar plots indicate the proportion of each subtype according to exposure to neoadjuvant chemotherapy (treatment status yes versus no).

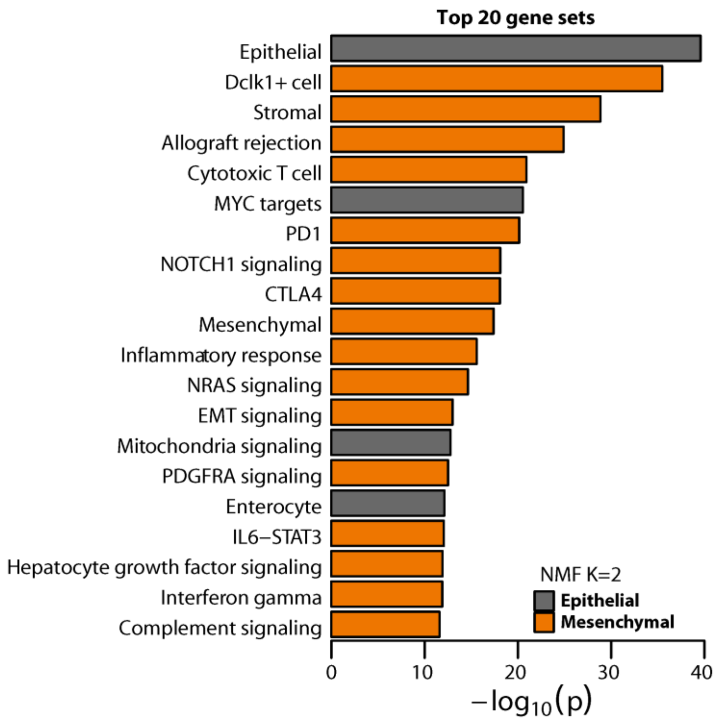

Fig. S5. GSEA of the epithelial versus mesenchymal subtype from K=2 factorization.

The plot shows the top 20 enriched pathways in the two subtypes as indicated by colors and ranked according to p-values from GSEA.

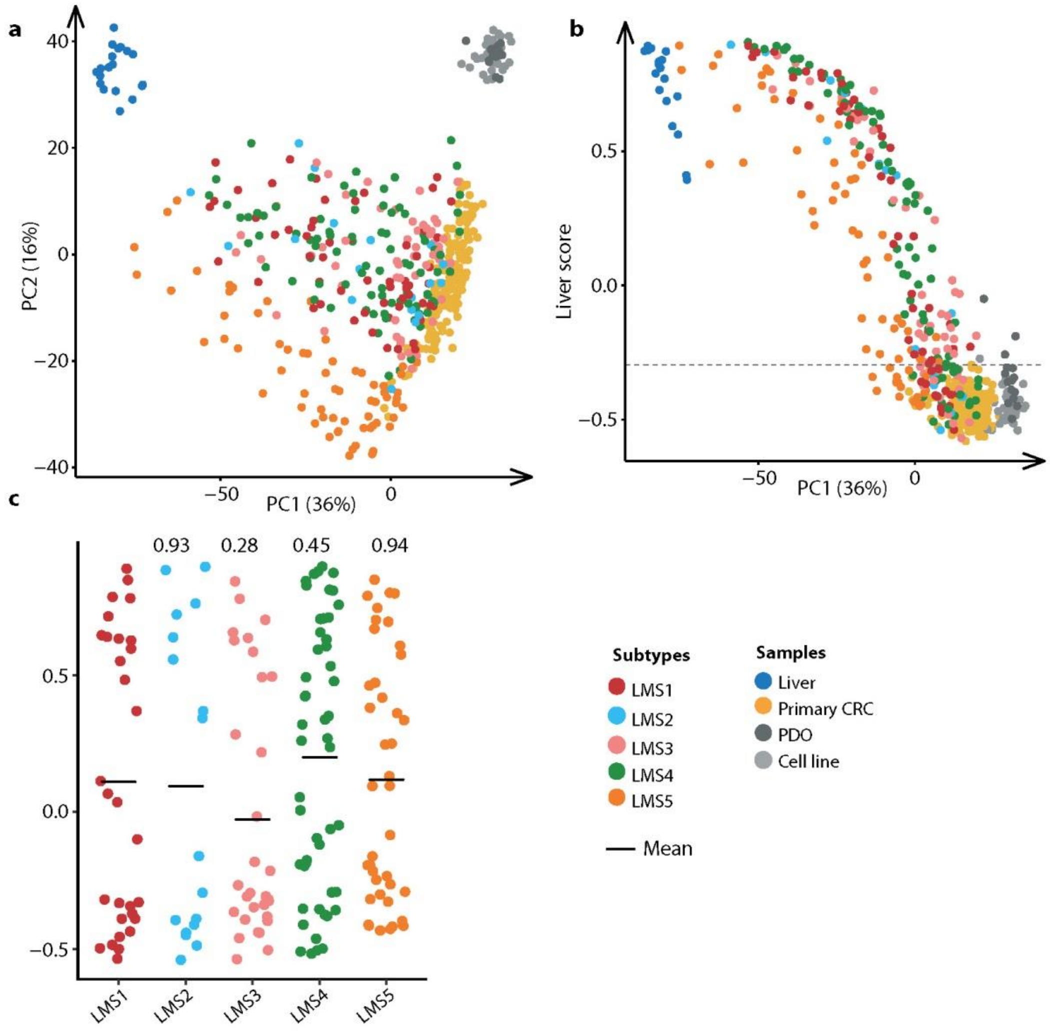

Fig. S6. PCA and liver score distribution among LMS groups.

a-b The same plots as Fig. 1**a** and 1**b**, respectively, with CRLM samples colored according to LMS. **c** Distribution of the "liver scores" among the LMS groups indicated no influence of the proportion of hepatocyte signals on de novo subtyping. Wilcoxon test p-values were calculated using LMS1 as reference group, analyzing single samples from each patient (n=169).

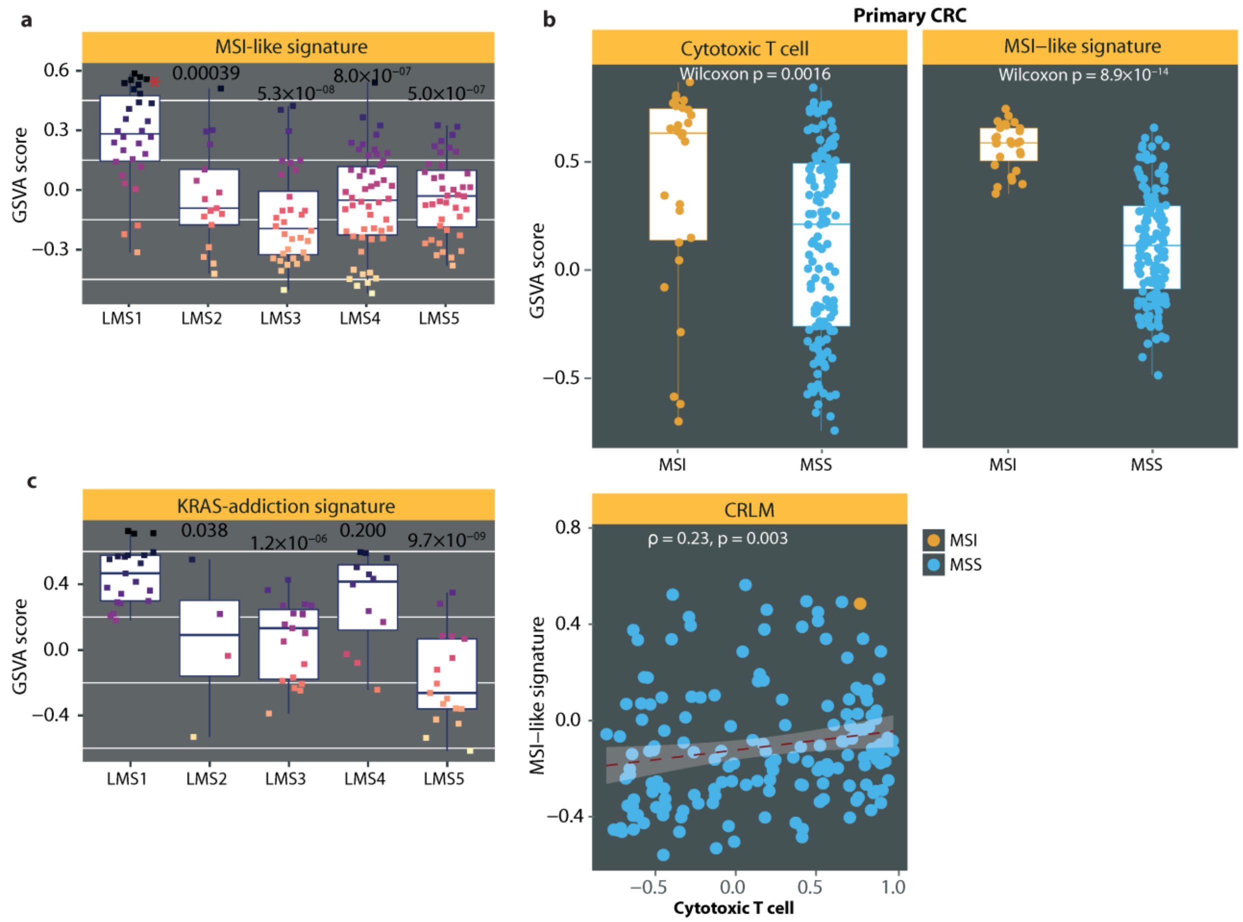

Fig. S7. Selected single-sample GSVA scores across the LMS groups.

a MSI-like signature score for each sample (one randomly selected per patient) across the LMS groups. Red asterisk denotes the single sample with confirmed MSI+ status. **b** Cytotoxic T cell and MSI-like scores plotted by MSI-status in primary CRCs illustrate the relationship between MSI and cytotoxic T cell infiltration in the primary setting. In contrast, the correlation between the MSI-like score and cytotoxic T cell score in CRLMs (mostly MSS) is weak. The linear smooth is represented by the red dashed line. The ρ value represents Spearman's correlation. **c** KRAS-addiction signature scores in CRLMs with confirmed *KRAS* mutation plotted according to LMS. Wilcoxon test p-values were calculated with LMS1 as reference group.

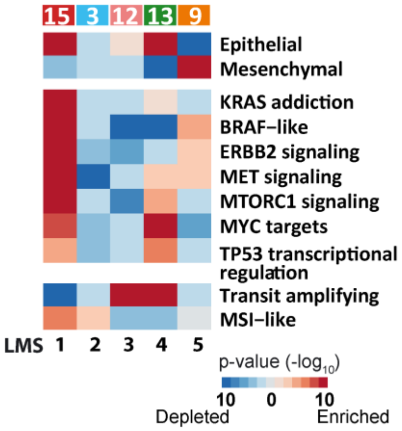

Fig. S8. GSEA in CRLMs with *RAS/TP53* co-mutations.

Oncogenic pathway enrichments were similar to results from GSEA of all samples. The color intensities represent p-values from comparison of one subtype against all others.

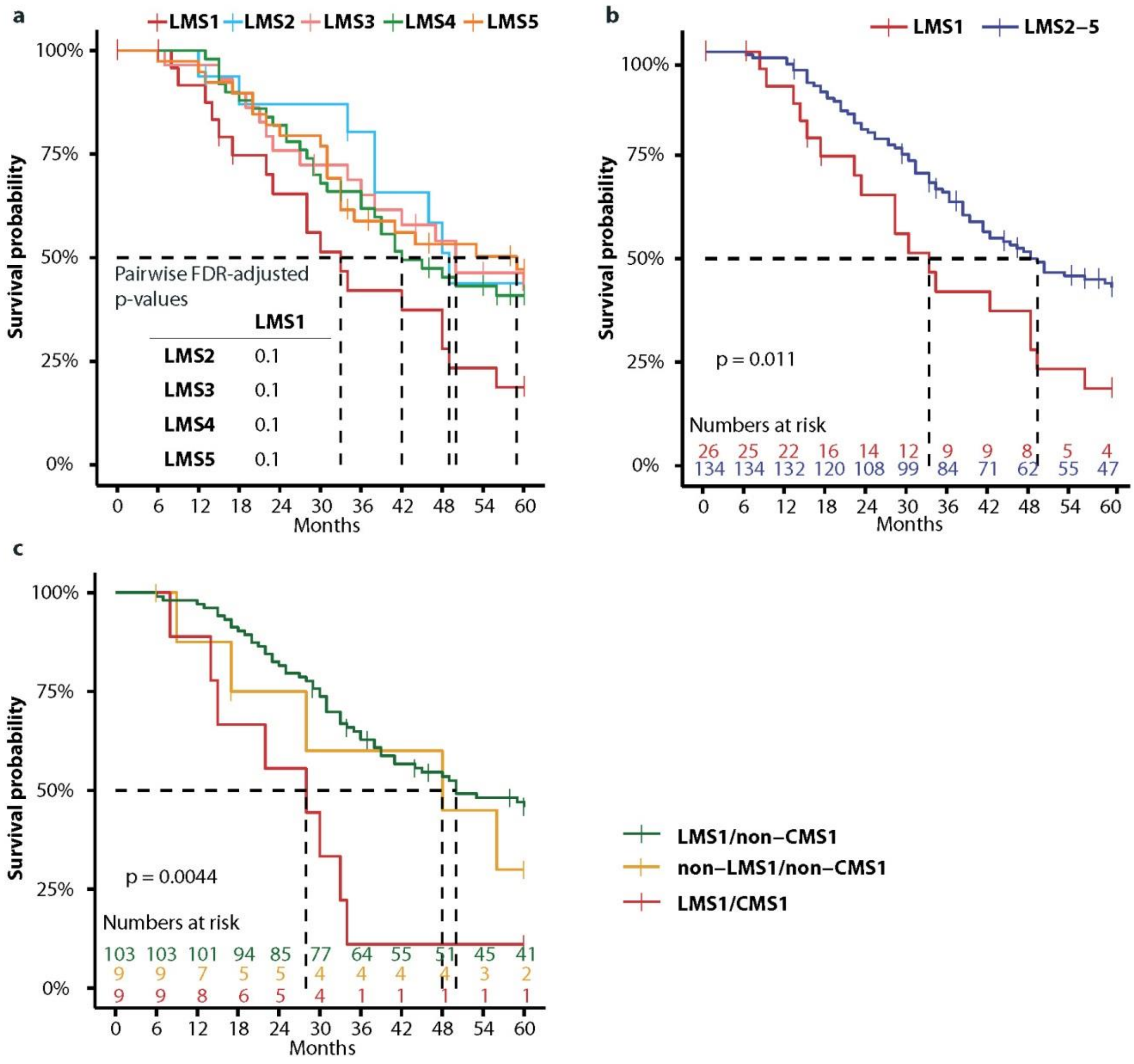

Fig. S9. Kaplan–Meier plots of 5-year CSS according to LMS and translated CMS subtypes.

Kaplan-Meier plots of 5-year CSS stratified by **a** individual LMS subtype, **b** LMS1 versus LMS2-5 grouped, and **c** according to both LMS1 and translated CMS subtypes. Patients with R2 resections in the liver were excluded. Log-rank test p-values are denoted (FDR corrected by the Benjamini-Hochberg in a).

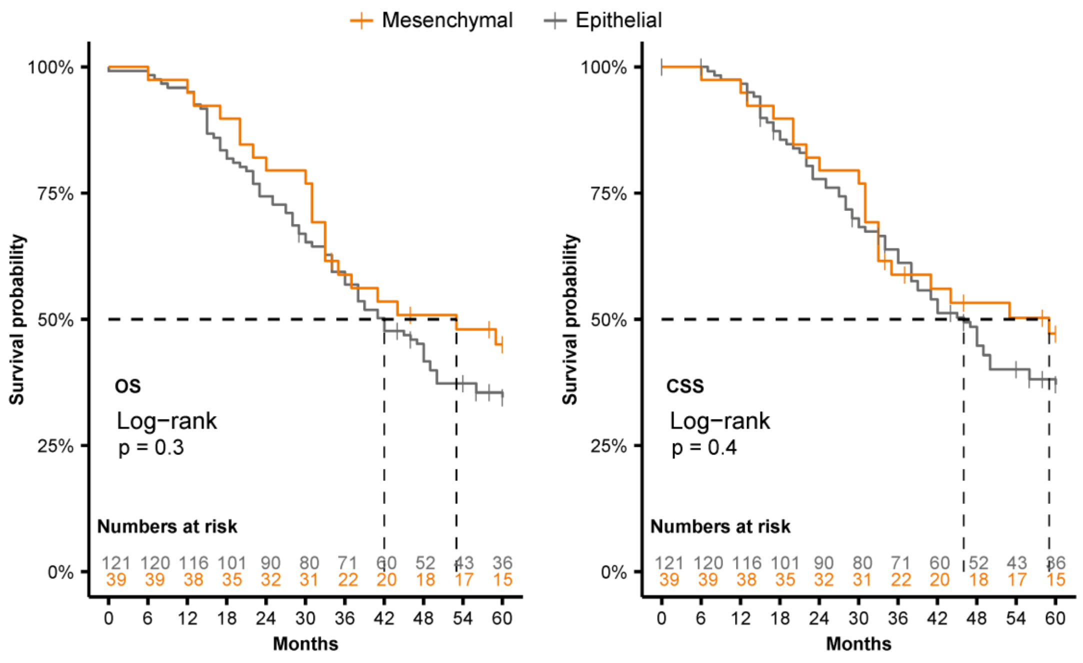

Fig. S10. Kaplan–Meier plots of 5-year OS and CSS according to epithelial and mesenchymal subtypes.

No difference in patient survival was observed when tumors were classified as epithelial or mesenchymal (result from NMF classification at K=2). Long-rank test p-values are denoted. Numbers at risk in each class are shown in the plot with the corresponding color. Patients with R2 resections in the liver were excluded.

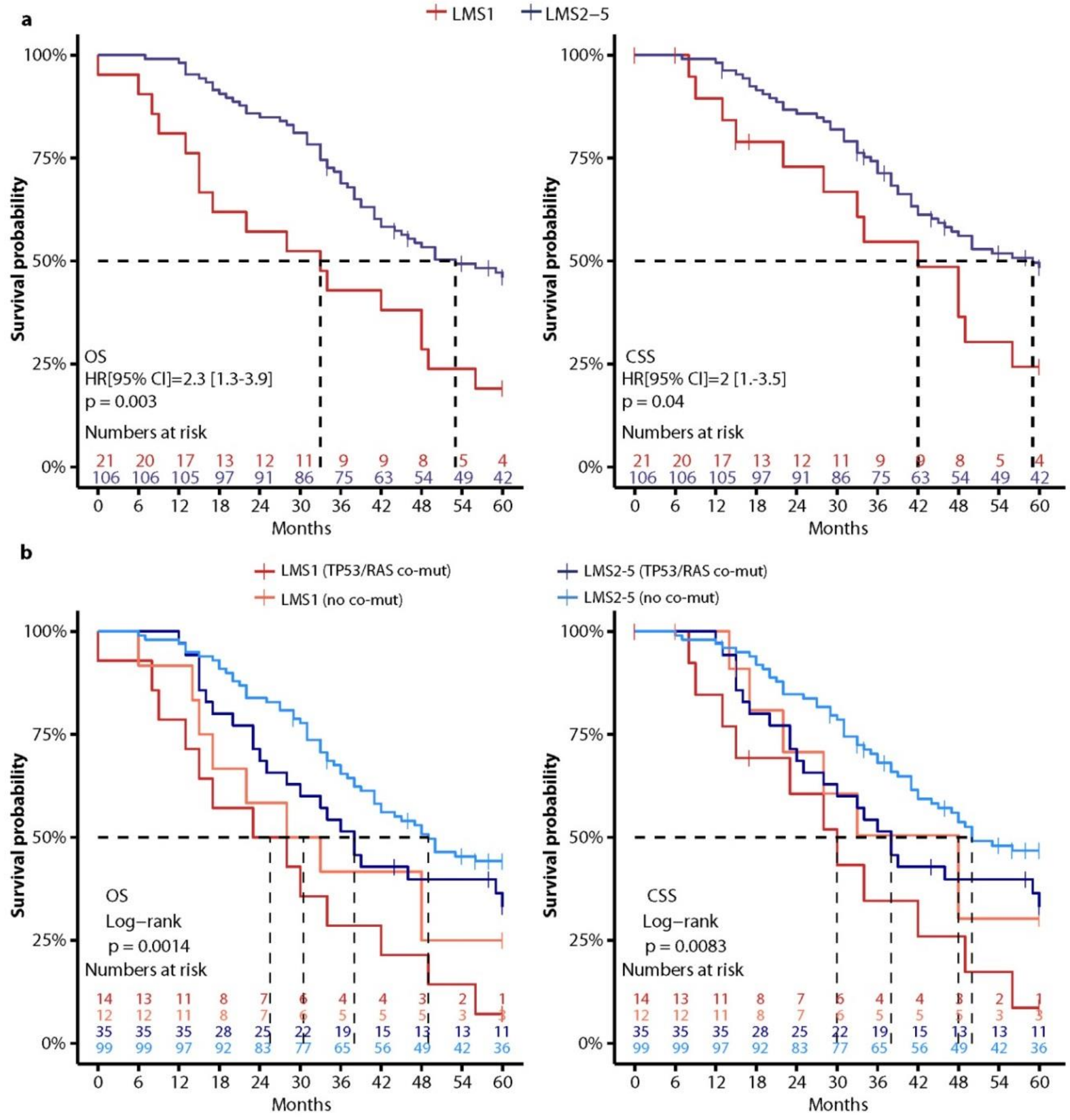

Pairwise FDR-adjusted p-values

OS	LMS1(co-mut)	LMS1(no co-mut)	LMS2-5(co-mut)
LMS1(no co-mut)	0.4	-	-
LMS2-5(co-mut)	0.08	0.5	-
LMS2-5(no co-mut)	0.0008	0.1	0.2

CSS	LMS1(co-mut)	LMS1(no co-mut)	LMS2-5(co-mut)
LMS1(no co-mut)	0.3	-	-
LMS2-5(co-mut)	0.2	0.9	-
LMS2-5(no co-mut)	0.006	0.3	0.2

Fig. S11. Kaplan–Meier plots of 5-year OS and CSS according to LMS and *TP53*/*RAS* co-mutations.

a LMS1 was associated with a poor patient outcome compared to LMS2-5 when analyzing only patients with R0/R1 resections (excluding both patients with R2 resection in the liver, and patients with extra hepatic disease, totally 42 patients). Long-rank test p-values are denoted. **b** Patient stratification according to both LMS1 versus LMS2-5 and *TP53*/*RAS* co-mutation versus no co-mutation showed that there was no significant difference for LMS1 tumors with and without co-mutations. Furthermore, LMS1

tumors with co-mutations were associated with a significantly worse 5-year OS than LMS2-5, both with and without co-mutations. A significant difference in the 5-year CSS rate was only observed in the comparison of co-mutated LMS1 versus LMS2-5 without co-mutation. Numbers at risk in each class are shown in the plot, with corresponding colors. Pairwise FDR-adjusted P-values from log-rank tests are denoted.

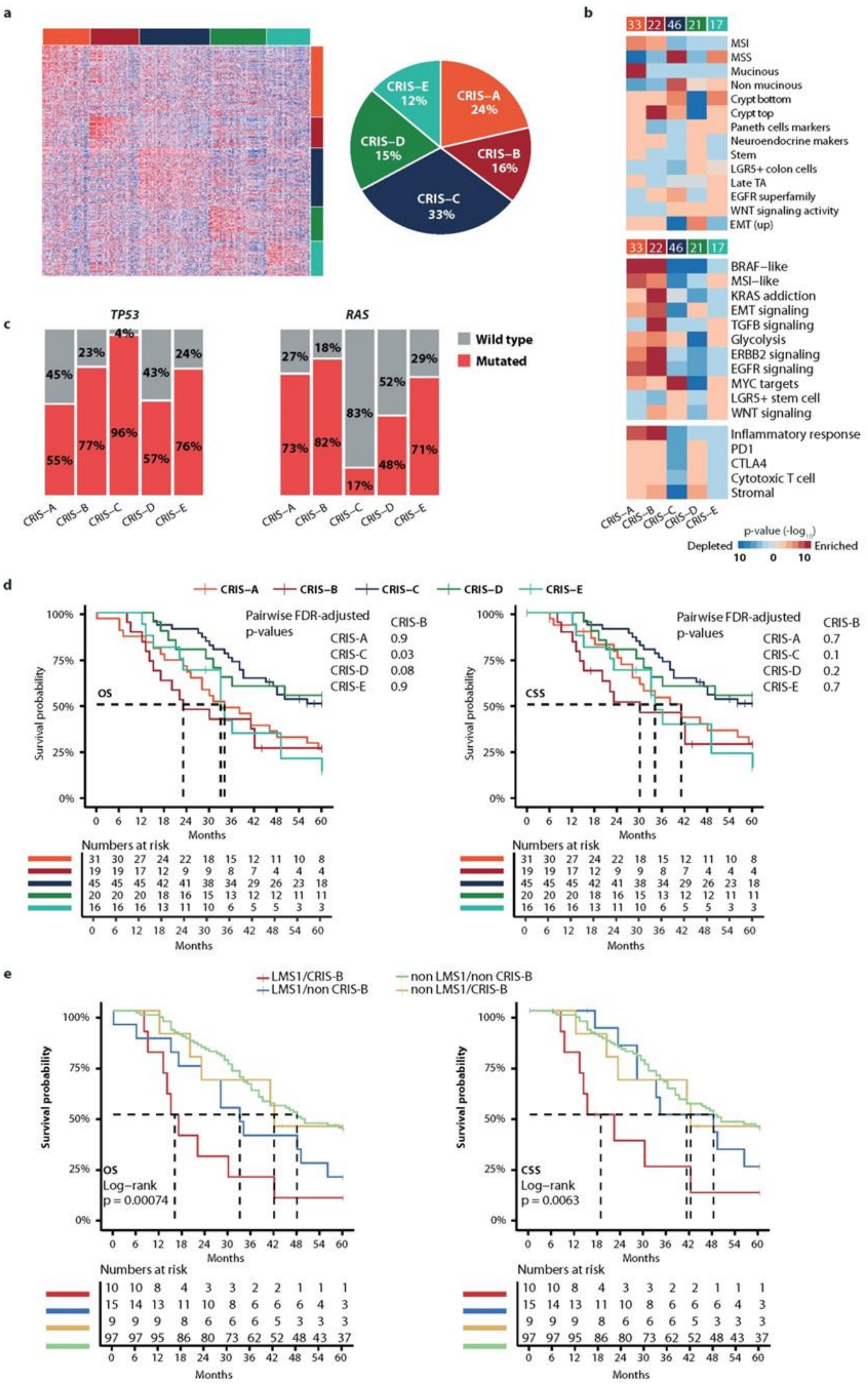
a
CRIS–A 24%
CRIS–B 16%
CRIS–C 33%
CRIS–D 15%
CRIS–E 12%
b
33 22 46 21 17
MSI
MSS
Mucinous
Non mucinous
Crypt bottom
Crypt top
Paneth cells markers
Neuroendocrine makers
Stem
LGR5+ colon cells
Late TA
EGFR superfamily
WNT signaling activity
EMT (up)
BRAF–like
MSI–like
KRAS addiction
EMT signaling
TGFB signaling
Glycolysis
ERBB2 signaling
EGFR signaling
MYC targets
LGR5+ stem cell
WNT signaling
Inflammatory response
PD1
CTLA4
Cytotoxic T cell
Stromal
CRIS–A CRIS–B CRIS–C CRIS–D CRIS–E
p-value (-log10)
Depleted Enriched
10 0 10
c
TP53
45% 23% 4% 43% 24%
55% 77% 96% 57% 76%
RAS
27% 18% 83% 52% 29%
73% 82% 17% 48% 71%
Wild type
Mutated
CRIS–A CRIS–B CRIS–C CRIS–D CRIS–E
d
CRIS–A CRIS–B CRIS–C CRIS–D CRIS–E
OS
Survival probability
100% 75% 50% 25% 0%
Pairwise FDR-adjusted p-values
CRIS-B
CRIS-A 0.9
CRIS-C 0.03
CRIS-D 0.08
CRIS-E 0.9
0 6 12 18 24 30 36 42 48 54 60
Months
Numbers at risk
31 30 27 24 22 18 15 12 11 10 8
19 19 17 12 9 9 8 7 4 4 4
45 45 45 42 41 38 34 29 26 23 18
20 20 20 18 16 15 13 12 12 11 11
16 16 16 13 11 10 6 5 5 3 3
CSS
Pairwise FDR-adjusted p-values
CRIS-B
CRIS-A 0.7
CRIS-C 0.1
CRIS-D 0.2
CRIS-E 0.7
e
LMS1/CRIS-B
LMS1/non CRIS-B
non LMS1/non CRIS-B
non LMS1/CRIS-B
OS
Log–rank
p = 0.00074
CSS
Log–rank
p = 0.0063
Numbers at risk
10 10 8 4 3 3 2 2 1 1 1
15 14 13 11 10 8 6 6 6 4 3
9 9 9 8 6 6 6 5 3 3 3
97 97 95 86 80 73 62 52 48 43 37

Fig. S12. CRIS classification of the in-house CRLM samples.

CRIS confidently classified 139 of the samples (82%, default threshold of FDR <0.2, one randomly selected samples per patient, n=169). **a** Heatmap represents the zscore of gene expression for gene templates (in rows) in each sample (in columns), both grouped according to the five CRIS classes. Red and blue indicate high and low relative expression of the genes, respectively. The sample subtype distribution is shown in the pie chart. **b** GSEA results from comparisons of the CRIS groups using two sets of gene signatures; top: signatures provided in the original CRIS paper; and bottom: in-house compiled gene sets. The color intensities represent p-values from comparison of one subtype against all others. This confirmed enrichment with MSI-like features and an inflammatory phenotype in CRIS-A and CRIS-B, as well as strong TGFβ activity in CRIS-B. **c** *TP53* and *RAS* mutation distribution in each subtype, confirming frequent *TP53* wild-type status in CRIS-A and CRIS-D, as well as frequent *RAS* wild-type status in CRIS-C. **d** Kaplan–Meier plots of 5-year overall survival (OS) and cancer-specific survival (CSS) according to the CRIS groups. The poor-prognostic CRIS-B group had a significantly lower survival rate compared to CRIS-C and CRIS-D only, but a similar survival rate as CRIS-A and CRIS-E. Pairwise FDR-adjusted P-values from log-rank tests are denoted. **e** Combined survival analyses of LMS and CRIS were focused on the LMS1 and CRIS-B groups, and LMS1 and CRIS-B combined was associated with the worst outcome, followed by LMS1/non-CRIS-B, non-LMS1/CRIS-B, and non-LMS1/non-CRIS-B. Patients with R2 resections in the liver were excluded from d and e. Numbers at risk in each class are shown and are indicated with corresponding colors.

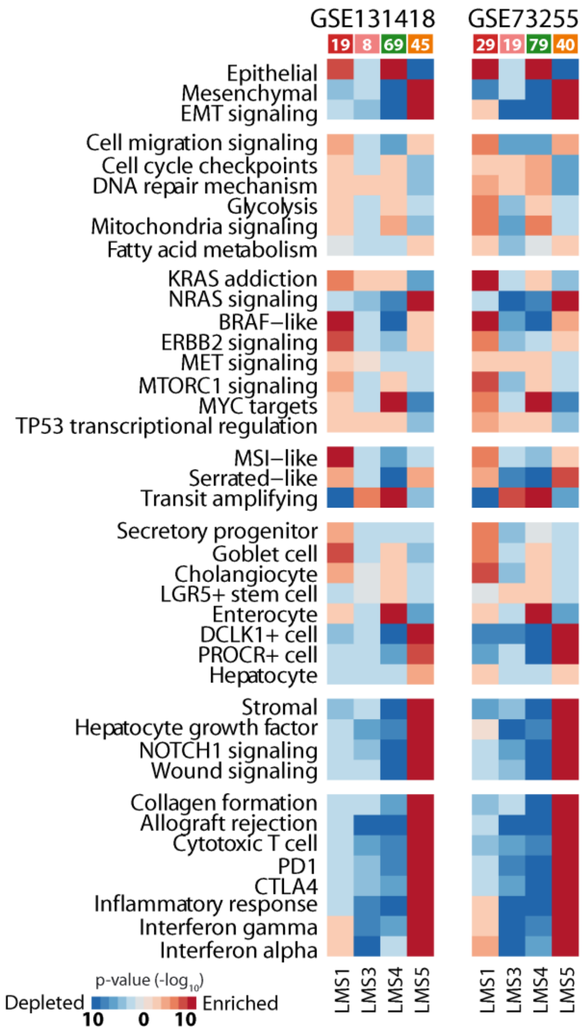

Fig. S13: GSEA of CRLMs in two external data sets according to LMS.

Enrichment patterns from GSEA were concordant between each of the two independent datasets and the in-house material (plot corresponding to Fig. 3**a**). The color intensities represent p-values from comparison of one subtype against all others.

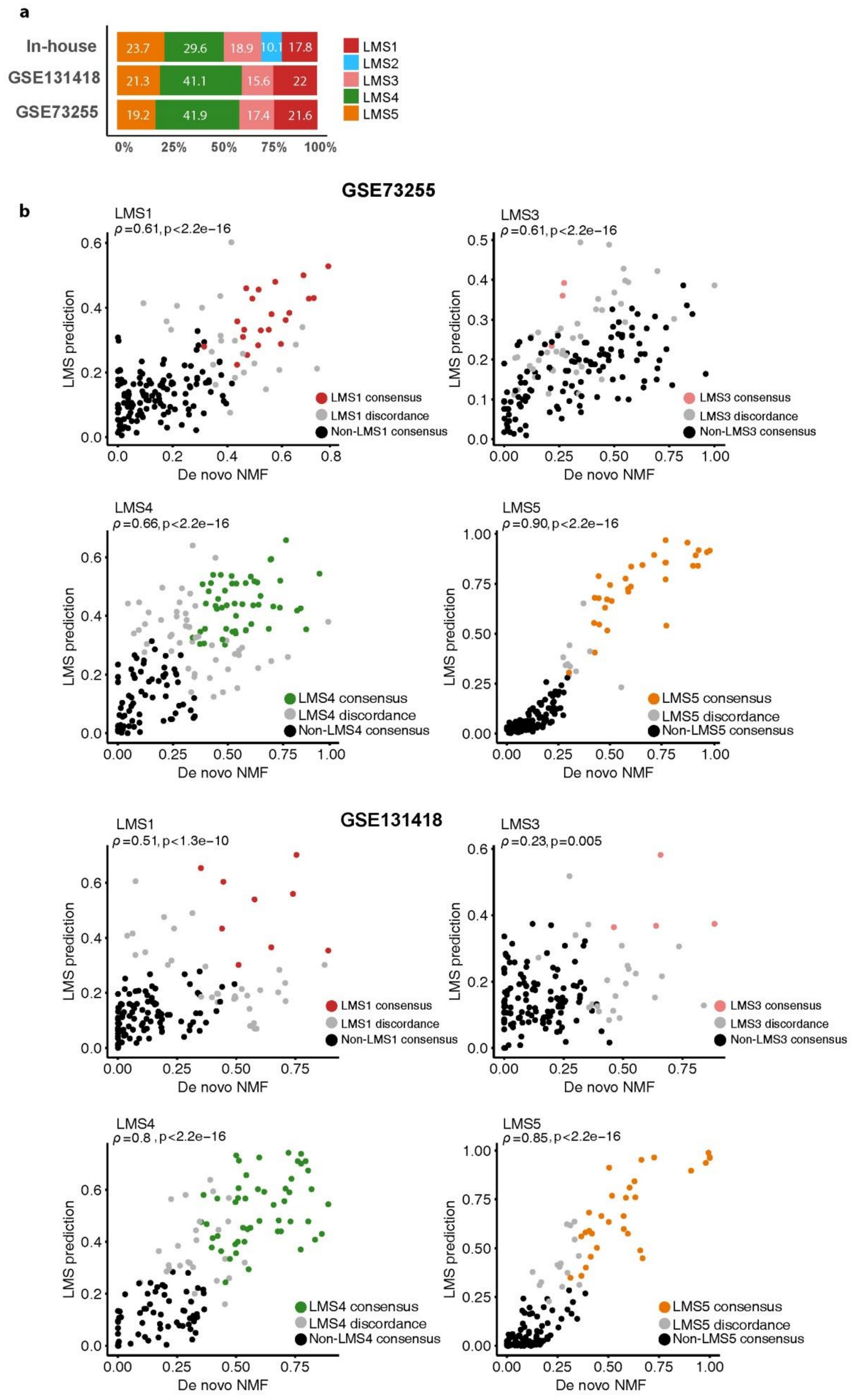
a
In-house
GSE131418
GSE73255
23.7
29.6
18.9
10.1
17.8
21.3
41.1
15.6
22
19.2
41.9
17.4
21.6
0%
25%
50%
75%
100%
LMS1
LMS2
LMS3
LMS4
LMS5
GSE73255
b
LMS1
ρ=0.61, p<2.2e−16
LMS1 consensus
LMS1 discordance
Non-LMS1 consensus
LMS3
ρ=0.61, p<2.2e−16
LMS3 consensus
LMS3 discordance
Non-LMS3 consensus
LMS4
ρ=0.66, p<2.2e−16
LMS4 consensus
LMS4 discordance
Non-LMS4 consensus
LMS5
ρ=0.90, p<2.2e−16
LMS5 consensus
LMS5 discordance
Non-LMS5 consensus
GSE131418
LMS1
ρ=0.51, p<1.3e−10
LMS1 consensus
LMS1 discordance
Non-LMS1 consensus
LMS3
ρ=0.23, p=0.005
LMS3 consensus
LMS3 discordance
Non-LMS3 consensus
LMS4
ρ=0.8 , p<2.2e−16
LMS4 consensus
LMS4 discordance
Non-LMS4 consensus
LMS5
ρ=0.85, p<2.2e−16
LMS5 consensus
LMS5 discordance
Non-LMS5 consensus
LMS prediction
De novo NMF

Fig. S14. De novo transcriptomic subtyping by unsupervised NMF of the two external CRLM datasets.

a Subtype distributions from de novo subtyping of the in-house (corresponding to the pie chart shown in main Fig. 2d) and two external CRLM datasets. LMS groups of the two external datasets were assigned based on the NMF cluster with the largest number of sample overlaps with supervised LMS predictions. **b** Comparisons of sample-wise posterior probabilities from the LMS random forest prediction model (vertical axes) and the de novo NMF subtypes (horizontal axes) in each external dataset. Samples with concordant classification between methods (consensus samples) are colored according to the LMS color scheme, or black, as indicated. Samples that are discordantly classified (subtype called by only one of the methods) are colored gray. Notably, each sample was assigned to the subtype with the maximum score for each of the supervised LMS and unsupervised classifications (no fixed threshold for classification).

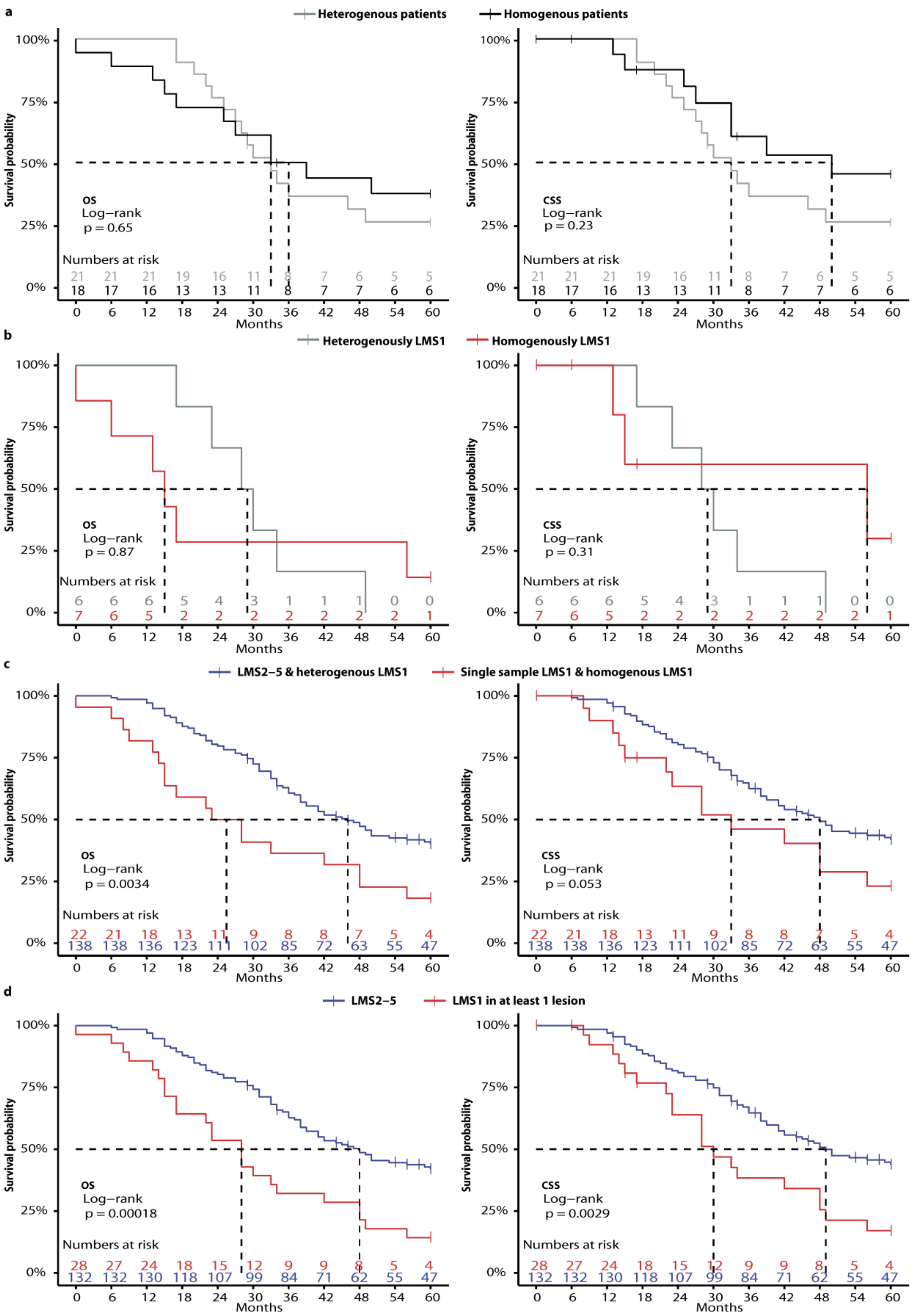
a
Heterogenous patients
Homogenous patients
OS
Log−rank
p = 0.65
CSS
Log−rank
p = 0.23
b
Heterogenously LMS1
Homogenously LMS1
OS
Log−rank
p = 0.87
CSS
Log−rank
p = 0.31
c
LMS2−5 & heterogenous LMS1
Single sample LMS1 & homogenous LMS1
OS
Log−rank
p = 0.0034
CSS
Log−rank
p = 0.053
d
LMS2−5
LMS1 in at least 1 lesion
OS
Log−rank
p = 0.00018
CSS
Log−rank
p = 0.0029
Survival probability
Numbers at risk
Months

Fig. S15. Kaplan–Meier curves of OS and CSS according to LMS1 and tumor heterogeneity.

Only patients with R0/R1 resections in the liver were included for analyses. There was no significant difference in the 5-year survival rates **a** between patients with homogenous versus heterogeneous LMS classifications in inter-metastatic comparisons; or **b** between patients homogenously classified with LMS1 in all samples versus patients with heterogeneous classifications including at least one LMS1 lesion/sample. LMS1 had poor prognostic associations independent of tumor heterogeneity, as shown by stratification of all patients according to **c** homogenous LMS1classification (LMS1 in all samples analyzed) versus LMS2-5 plus heterogeneous LMS1 classification; and **d** LMS1 in at least one lesion versus LMS2-5 in all lesions. Long-rank test p-values are shown. Numbers at risk in each class are shown in the plot, with the corresponding color.

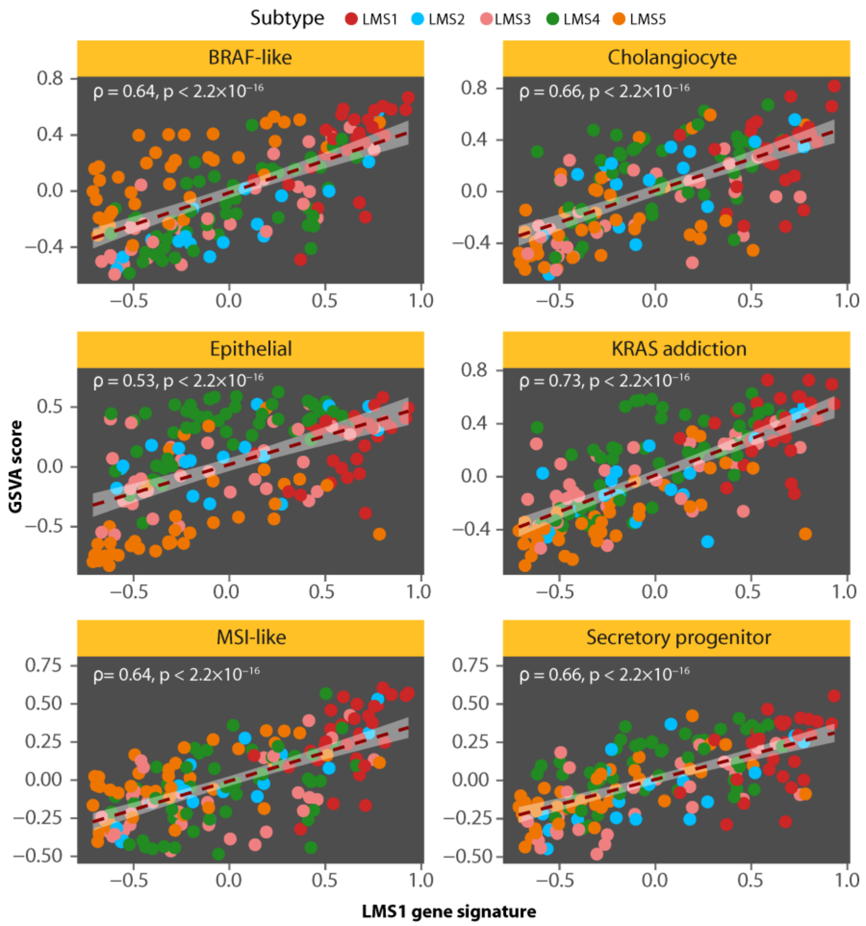

Fig. S16. LMS1 mini-classifier is correlated with signatures of LMS1 characteristics.

Cross-sample comparisons (single samples from each of the 169 patients in the in-house series) of GSVA scores for the 9 genes in the LMS1 mini-classifier (horizontal axes) and several pathways enriched in LMS1 (vertical axes), as indicated. The rho value represents Spearman's correlation.

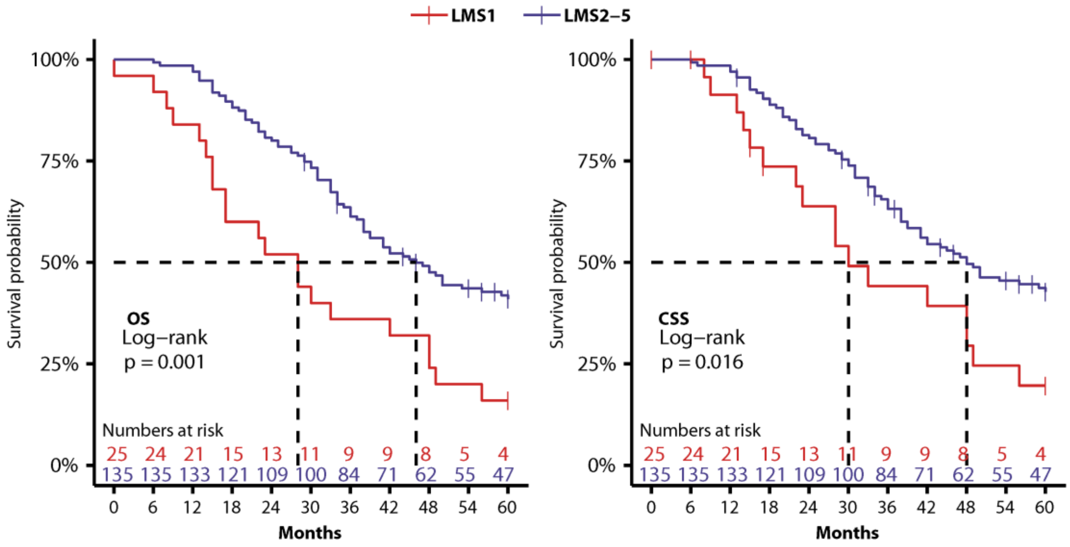

Fig. S17. LMS1 mini-classifier captures the poor-prognostic value of LMS1.

Kaplan-Meier plots of 5-year OS and CSS for patient stratification based on the LMS1 mini-classifier. Patients with R2 resections in the liver are excluded. Numbers at risk in each class are shown in the plot, with corresponding colors.
